# Uncovering direct and indirect molecular determinants of chromatin loops using a computational integrative approach

**DOI:** 10.1371/journal.pcbi.1005538

**Published:** 2017-05-23

**Authors:** Raphaël Mourad, Lang Li, Olivier Cuvier

**Affiliations:** 1 Laboratoire de Biologie Moléculaire Eucaryote (LBME), CNRS, Université Paul Sabatier (UPS), Toulouse, France; 2 Center for Computational Biology and Bioinformatics (CCBB), Indiana University, Indianapolis, Indiana, United States of America; Rutgers University, UNITED STATES

## Abstract

Chromosomal organization in 3D plays a central role in regulating cell-type specific transcriptional and DNA replication timing programs. Yet it remains unclear to what extent the resulting long-range contacts depend on specific molecular drivers. Here we propose a model that comprehensively assesses the influence on contacts of DNA-binding proteins, cis-regulatory elements and DNA consensus motifs. Using real data, we validate a large number of predictions for long-range contacts involving known architectural proteins and DNA motifs. Our model outperforms existing approaches including enrichment test, random forests and correlation, and it uncovers numerous novel long-range contacts in *Drosophila* and human. The model uncovers the orientation-dependent specificity for long-range contacts between CTCF motifs in *Drosophila*, highlighting its conserved property in 3D organization of metazoan genomes. Our model further unravels long-range contacts depending on co-factors recruited to DNA indirectly, as illustrated by the influence of cohesin in stabilizing long-range contacts between CTCF sites. It also reveals asymmetric contacts such as enhancer-promoter contacts that highlight opposite influences of the transcription factors EBF1, EGR1 or MEF2C depending on RNA Polymerase II pausing.

## Author summary

Chromosomal DNA is tightly packed in three dimensions (3D) such that a 2-meter long human genome can fit into a microscopic nucleus. Recent studies have revealed that such packing of DNA is not random but instead structured into functional DNA loops. Those loops are essential to numerous key processes in the cell, such as genome expression and DNA replication. In addition, disruption of DNA loops can lead to genetic diseases and cancers. Understanding how DNA loops are formed and what are their molecular determinants is thus a fundamental issue. In this work, we propose a computational model to identify the molecular determinants of loops, including protein and DNA sequence. Most notably, the model offers insights in the different mechanistic scenarios behind loop formation. Using this model, we uncover numerous novel DNA loops and underlying mechanisms in *Drosophila* and human. We find that the orientation-dependent specificity between CTCF motifs is conserved in metazoans. We show how loops between DNA-binding proteins can be mediated by additional cofactors. Our analyses further reveal opposite influences of transcription factors depending on RNA Polymerase II pausing.

## Introduction

Chromosomal DNA is tightly packed in three dimensions (3D) such that a 2-meter long human genome can fit into a nucleus of approximately 10 microns in diameter [1]. Such 3D structure of chromosome has recently been explored by chromosome conformation capture combined with high-throughput sequencing technique (Hi-C) at an unprecedented resolution [2–4]. Multiple hierarchical levels of genome organization have been uncovered such as compartments A/B [5] and topologically associating domains (TADs) [2, 3]. In particular, TADs represent a pervasive structural feature of the genome organization and are highly conserved across species. Functional studies revealed that spatial organization of chromosome is essential to numerous key processes such as for the regulation of gene expression by distal enhancers [4] or for the replication-timing program [6].

The comprehensive analysis of 3D chromatin drivers is currently a hot topic [7]. A growing body of evidence supports the role of insulator binding proteins (IBPs) such as CTCF, and cofactors like cohesin, as mediators of long-range chromatin contacts [3, 8, 9]. In human, high-resolution Hi-C mapping has recently revealed that loops that demarcate domains were often marked by asymmetric CTCF motifs where cohesin is recruited [10]. Depletions of CTCF and cohesin decreased chromatin contacts [11]. However the impact of these depletions was limited suggesting that other proteins might be involved in shaping the chromosome in 3D. For instance, numerous IBPs, cofactors and functional elements were shown to colocalize at TAD borders [9, 12]. The identification of 3D chromatin drivers is thus an active avenue of research. Computational approaches that integrate the large amount of available protein binding data (chromatin immunoprecipitation followed by high-throughput DNA sequencing, ChIP-seq), functional elements (promoters and enhancers), and DNA motifs, with Hi-C data may be well-suited to identify novel factors that participate in shaping the chromosome in 3D [13].

In this paper, we propose a model to comprehensively analyze the roles of genomic features, such as DNA-binding proteins or motifs, in establishing or maintaining chromatin contacts. The proposed model offers insights in the different mechanistic scenarios behind loop formation, because of its ability to rigorously assess the effect of protein complex on long-range contact frequency. Using real data, the model successfully predicted numerous long-range interactions involving motifs and proteins as highlighted in previous independent studies. Moreover, our model outperformed current approaches to identify architectural proteins and motifs, and to detect the effects of single nucleotide polymorphisms (SNPs) in the dCTCF motif. In addition, our model is the only approach able to assess the effect of a cofactor in mediating long-range contacts between distant protein binding sites, such as cohesin with CTCF. Using recent *Drosophila* and human Hi-C data at high resolution, combined with a large number of ChIP-seq, RNA-seq, CAGE-seq and DNA motif data, we revealed numerous novel motifs, insulator binding proteins, cofactors and functional elements that positively or negatively impact long-range contacts depending on transcriptional activity or motif orientation.

### Results and discussion

### The model

We propose to use a generalized linear model with interactions (GLMI) to analyze the effects of genomic features such as architectural protein co-occupancies on chromatin contacts at genome-wide level:

$$\begin{array}{lll} \text{log}\left(\text{E}\left[y|X\right]\right) & = & \beta_{0}+\beta X\\ & = & \beta_{0}+\beta_{d}d+\beta_{B}B+\beta_{C}C+\beta_{g}g \end{array}$$ (1)

Variable **y** denotes the number of Hi-C contacts for any pair of bins on the same chromosome. Variable set **X** = {**d**, **B**, **C**, **g**} comprises several variable subsets: the log-distance variable **d**, the bias variables **B**, the confounding variable set **C** and the genomic variable of interest **g**. The log-distance variable **d** accounts for the background polymer effect (log-log relation between distance and Hi-C count) [14]. Bias variables **B** = {**len**, **GC**, **map**} are known Hi-C biases including fragment length (**len**), GC-content (**GC**) and mappability (**map**) that are computed as in [15] (S1 Appendix, Bias variable computation). Including those bias variables into the model allows to correct for biases in Hi-C data. Bias normalization by matrix balancing methods [16] is avoided, because these methods might remove effect of genomic variable of interest. Variable **g** represents the genomic feature of interest, whose associated *β*_*g*_ parameter value reflects its effects on chromatin contacts. Variable set **C** comprises confounding variables included to properly estimate *β*_*g*_. Model (1) is very general and can be developed in multiple versions depending on the variable **g** of interest. In the following paragraphs, we will see the different kinds of variables **g**. The corresponding models are detailed in Subsection Materials and Methods, The different models.

**Fig 1:**
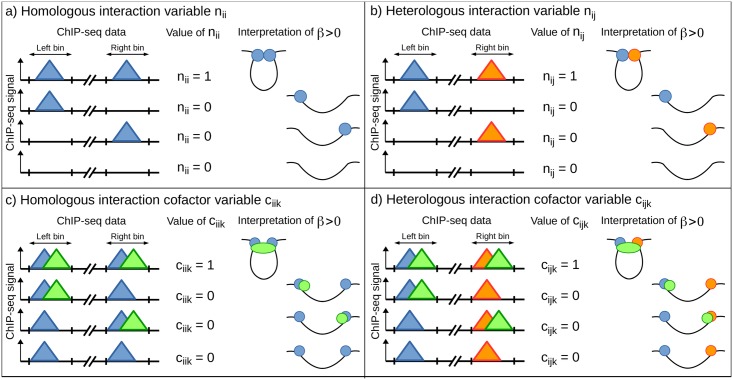
Illustration of the proposed model and variables in the context of protein ChIP-seq data. a) Homologous interaction variable. b) Heterologous interaction variable. c) Homologous interaction cofactor variable. d) Heterologous interaction cofactor variable. The 3 proteins *F*_*i*_, *F*_*j*_ and *F*_*k*_ are colored in blue, red and green, respectively. Here *F*_*i*_ and *F*_*j*_ are insulator binding proteins (IBPs), and *F*_*k*_ is a cofactor (recruited by IBPs).

We illustrate the different model variables in Fig 1. For simplicity, we illustrate our model with protein binding sites, yet the same model is applicable to many other genomic features such as motifs or promoters. Let consider a pair of bins that we call left bin (L) and right bin (R). The attribution for left and right bins is arbitrary. Let also consider 3 genomic features *F*_*i*_ (whose binding is colored in blue in Fig 1), *F*_*j*_ (in red) and *F*_*k*_ (in green) that represent binding sites of 3 different proteins. For the genomic feature *F*_*i*_, occupancy variables **z**_*iL*_ and **z**_*iR*_ denote the occupancies of *F*_*i*_ on left and right bins, respectively. For an occupancy variable, a value of 0/1 means absence/presence of the corresponding feature on the bin, *e.g.* absence/presence of the protein on the bin (a value between 0 and 1 means partial overlap of the feature). Occupancy variables are used to build 4 main kinds of model variables as follows.

A “homologous interaction” variable **n**_*ii*_ is the product of **z**_*iL*_ and **z**_*iR*_ (**n**_*ii*_ = **z**_*iL*_ × **z**_*iR*_). The associated $\beta_{n_{ii}}$ parameter reflects the extent by which the genomic feature *F*_*i*_ interacts with itself through chromatin contacts (Fig 1a). For instance, distant CTCF binding sites were shown to form loops in human [10, 17].

A “heterologuous interaction” variable **n**_*ij*_ is the average of the product **z**_*iL*_ × **z**_*jR*_ and the product **z**_*jL*_ × **z**_*iR*_ ($n_{ij}=\frac{1}{2}\left(z_{iL}\times z_{jR}+z_{jL}\times z_{iR}\right)$), because both products are identically associated to **y**. The associated $\beta_{n_{ij}}$ parameter reflects the extent by which the genomic feature *F*_*i*_ interacts with another genomic feature *F*_*j*_ through chromatin contacts (Fig 1b). For instance, enhancers are in long-range contacts with promoters to regulate target gene expression [14, 18].

A “homologous interaction cofactor” variable **c**_*iik*_ is the product of an interaction variable **n**_*ii*_ and an interaction variable **n**_*kk*_ (**c**_*iik*_ = **n**_*ii*_ × **n**_*kk*_ = **z**_*iL*_ × **z**_*iR*_ × **z**_*kL*_ × **z**_*kR*_). Here we consider the cofactor *F*_*k*_ as a protein that does not directly bind to DNA, but which is instead bound by an insulator binding protein *F*_*i*_ (IBP) to DNA, such as cohesin is recruited by CTCF to DNA. Hence we expect that a cofactor will be found at both bins L and R in contact, *e.g.* cohesin ring entraps both chromatin fibers and is thus observed at both bins [10, 17]. That explains why **c**_*iik*_ is the product of **n**_*ii*_ and **n**_*kk*_. The associated $\beta_{c_{iik}}$ parameter reflects the extent by which chromatin contacts between genomic feature *F*_*i*_ and itself are mediated by a genomic feature *F*_*k*_, the cofactor (Fig 1c).

A “heterologous interaction cofactor” variable **c**_*ijk*_ is the product of an interaction variable **n**_*ij*_ and an interaction variable **n**_*kk*_ ($c_{ijk}=n_{ij}\times n_{kk}=\frac{1}{2}\left(z_{iL}\times z_{jR}\times z_{kL}\times z_{kR}+z_{jL}\times z_{iR}\times z_{kL}\times z_{kR}\right)$). Here we consider the cofactor *F*_*k*_ as a protein that does not directly bind to DNA, but which is instead bound to two IBPs *F*_*i*_ and *F*_*j*_. For instance, a loop can be mediated by CP190 that binds to BEAF-32 and GAF sites that are distant [19]. The associated $\beta_{c_{ijk}}$ parameter reflects the extent by which chromatin contacts between genomic features *F*_*i*_ and *F*_*j*_ are mediated by a third genomic feature *F*_*k*_, the cofactor (Fig 1d).

In the previous paragraphs, we introduced numerous variables that were the products of simpler variables, namely the occupancy variables. In (generalized) linear regression, those product variables are called “interaction” terms. To detect such interaction effects, one usually needs a large number of observations. We will see in the next subsections that the tremendous amount of data provided by Hi-C experiments allows to detect such interaction effects with accuracy. The model and the different variables will be illustrated with real world scenarios in the next subsections.

### Prediction of known factors and validation with experimental data

We first sought to validate our model using experimental data. For this purpose, we focused on the *Drosophila* model because several insulator binding proteins (IBPs) that mediate long-range interactions have been well characterized in this organism. *Drosophila* IBPs comprise suppressor of hairy wing (Su(Hw)), *Drosophila* CTCF (dCTCF), boundary-element-associated factor of 32 kDa (BEAF-32), GAGA binding factor (GAF), Zeste-White 5 (ZW5) [20], the general transcription factor dTFIIIC [9] and DNA replication-related element factor (DREF) [7]. We analyzed Kc167 Hi-C data at 10 kb resolution and focused on 20kb-1Mb distances for which contact frequencies were accurately measured experimentally [21]. At this distance range, the log-log relation between Hi-C count and distance was linear (*R*^2^ = 0.99, S1 Fig), supporting the use of the log-distance term in the model. The data comprised approximately 1 million of observations, which allowed to detect higher-order interactions with enough precision (tight parameter confidence intervals reflected by low p-values, see below). Because of Hi-C count overdispersion, we used negative binomial regression as the most appropriate specification of the generalized linear model.

**Fig 2:**
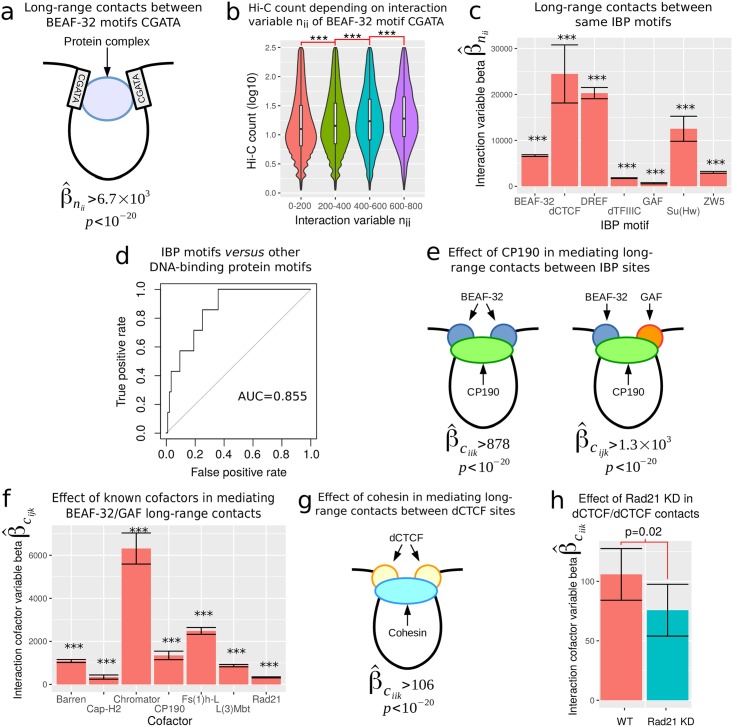
Biological validation of the model. a) Long-range contacts between BEAF-32 motifs. b) Hi-C count as a function of interaction variable **n**_*ii*_ of BEAF-32 motifs. c) Long-range contacts between same insulator binding protein (IBP) motifs. d) Receiver operating characteristic (ROC) curves of long-range contacts between same motifs. Known IBP motifs (true positives) are compared to other protein motifs (false positives). e) Effect of CP190 in mediating long-range contacts between IBP sites. f) Effect of known cofactors in mediating long-range contacts between distant BEAF-32 and GAF binding sites. Barren, Cap-H2 and Rad21 are subunits of condensin I, condensin II and cohesin, respectively. g) Effect of cohesin in mediating long-range contacts between dCTCF sites. h) Effect of cohesin in mediating long-range contacts between distant dCTCF binding sites in wild-type (WT) compared to Rad21 KD cells.

It has been shown that BEAF-32 motifs can form long-range interactions with each other using both fluorescence cross-correlation spectroscopy [22] and high-resolution microscopy [23]. Following this observation, we first validated our model by successfully estimating long-range contacts between the BEAF-32 CGATA motifs using model (2) (${\hat{\beta }}_{n_{ii}}=6.7\times 10^{3}$, *p* < 10^−20^; Fig 2a; model (2) and all other models used in the following are described in Subsection Materials and Methods, The different models). This result was confirmed as we observed that the Hi-C count increased with co-occupancy of BEAF-32 motifs (variable **n**_*ii*_) (Fig 2b). We also observed long-range contacts between dCTCF motifs (${\hat{\beta }}_{n_{ii}}=2.4\times 10^{4}$, *p* = 3 × 10^−14^), highlighting their important roles in loop formation in *Drosophila* as observed in human [10, 17]. Over the 7 known IBPs, the model correctly identified all IBP motifs as involved in long-range contacts among themselves (Fig 2c). Next the same approach was used to evaluate the model’s ability to discriminate between the 7 IBP motifs (true positives) and 83 other DNA-binding protein motifs (false positives). This approach obtained good predictions (area under the curve (AUC) = 0.855; Fig 2d). Among the motifs that we considered as false positives, M1BP and Ttk69K motifs presented high and significant interaction effects (M1BP: ${\hat{\beta }}_{n_{ii}}=1.7\times 10^{5}$; Ttk69K: ${\hat{\beta }}_{n_{ii}}=2.3\times 10^{4}$, *p* < 10^−12^, resp.). These results suggested that M1BP and Ttk69K might represent new insulator-binding protein candidates. Accordingly, M1BP protein binds to the promoters of paused genes that were shown to be involved in long-range contacts [18, 24]. Ttk69K protein has a homomeric dimerization BTB/POZ domain that could help bridging two distant proteins through long-range contacts [22].

We then used GLMI to study the role of cofactors that cannot directly bind to DNA, but are instead recruited by IBPs, and are required to mediate or stabilize long-range contacts between two IBP binding sites. In *Drosophila*, well-known cofactors include condensin I, condensin II, Chromator, centrosomal protein of 190 kDa (CP190), cohesin [19–22], Fs(1)h-L [25] and lethal (3) malignant brain tumor (L(3)Mbt) [7]. Most notably, fluorescence cross-correlation spectroscopy (FCCS) experiments have shown that CP190 is required to bridge long-range contacts between two BEAF-32 binding sites [22]. Using ChIP-seq peak data with model (4), we estimated a significant and positive effect of CP190 in mediating long-range contacts between BEAF-32 sites (${\hat{\beta }}_{c_{iik}}=878$, *p* < 10^−20^; Fig 2e), in complete agreement with recent work [22]. Similar result was obtained for Chromator in mediating long-range contacts between BEAF-32 sites (${\hat{\beta }}_{c_{iik}}=3.4\times 10^{3}$, *p* < 10^−20^) [22]. In addition, previous BEAF-32 mutation by our group has revealed that cofactor CP190 is also required to bridge long-range contacts between BEAF-32 and GAF binding sites [19]. Using ChIP-seq peak data with model (5), we estimated a significant and positive effect of CP190 in bridging distant BEAF-32 and GAF sites (${\hat{\beta }}_{c_{ijk}}=1.3\times 10^{3}$, *p* < 10^−20^; Fig 2e) [19]. We applied the same modeling approach to the 6 other known cofactors and found that all were associated with significant positive effects in mediating contacts between BEAF-32 and GAF binding sites (all betas ${\hat{\beta }}_{c_{ijk}}>326$, all p-values *p* < 10^−20^; Fig 2f). Because CP190 was also shown to mediate long-range contacts between BEAF-32 and dCTCF, and between BEAF-32 and Su(Hw) [19], we estimated the corresponding cofactor effects. We again found significant positive effect of CP190 between BEAF-32 and dCTCF (${\hat{\beta }}_{c_{ijk}}=892$, *p* < 10^−20^), but our method only detected a slightly significant mediating effect of CP190 between BEAF-32 and Su(Hw) (${\hat{\beta }}_{c_{ijk}}=175$, *p* = 0.02). In human, the most studied cofactor is cohesin that is able to entrap two chromatin fibers thereby stabilizing long-range contacts between CTCF sites [10, 17]. Hence we assessed the impact of cohesin in mediating long-range contacts between two dCTCF binding sites in *Drosophila*. We found a significant and positive effect of cohesin (${\hat{\beta }}_{c_{iik}}=105.8$, *p* < 10^−20^; Fig 2g), thus supporting a conserved function of cohesin in stabilizing long-range contacts between CTCF sites in metazoans.

We further tested our model for cofactor effects using perturbed conditions such as the removal of these cofactors, as obtained through knocking-down (KD) followed by Hi-C experiment. Of note, Hi-C experiments are expensive and complex to carry out, and the possibility to predict long-range contacts upon such KD is of major importance. We compared the impact of cohesin in the context of long-range contacts bridging CTCF sites in WT and Rad21 (cohesin subunit) KD Hi-C data. Our model estimated a significant but lower cofactor effect of cohesin in Rad21 KD (${\hat{\beta }}_{c_{iik}}=75.7$, *p* = 9 × 10^−12^), compared to WT (${\hat{\beta }}_{c_{iik}}=105.8$, *p* < 10^−20^). The difference between WT and Rad21 KD associated coefficients was negative and significant (beta difference = −30.1, *p* = 0.027), corresponding to a beta decrease of 28% (Fig 2h). This result therefore validated the estimated effect of cohesin in mediating distant dCTCF binding sites, which decreased upon cohesin depletion as expected.

Using real data, we concluded that our model successfully predicted the roles of IBP motifs in long-range contacts between distant loci, as well as the roles of known cofactors in bridging distant IBP binding sites. The GLMI predictions were validated in the literature and using protein KD followed by Hi-C experiment.

### GLMI outperformed existing methods

**Fig 3:**
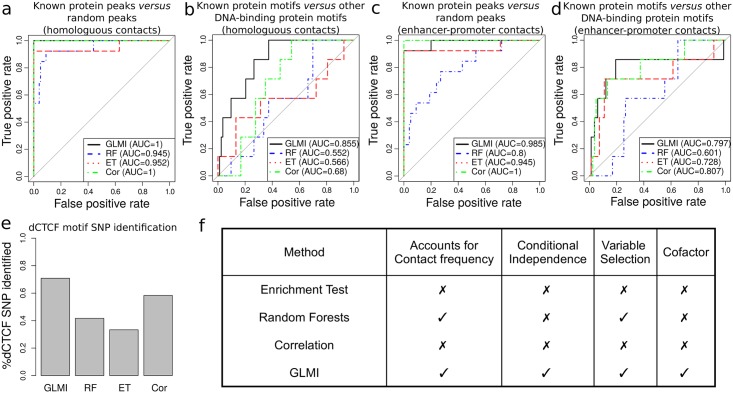
Comparisons between generalized linear regression with interactions (GLMI), highly confident chromatin interaction pair detection followed by pair type enrichment (ET), highly confident chromatin interaction pair detection followed by correlation (Cor) and random forests (RF). a) Receiver operating characteristic (ROC) curves of the four methods to distinguish between known protein peaks (13 true positives) and random peaks (100 false positives). Long-range contacts are assessed between a protein and itself (homologous contacts). b) ROC curves of the four methods to distinguish between known protein motifs (7 true positives) and other DNA-binding protein motifs (83 false positives). Long-range contacts are assessed between a motif and itself (homologous contacts). c) ROC curves of the four methods to distinguish between known protein peaks and random peaks. Long-range contacts are assessed between a protein and promoters (enhancer-promoter contacts). d) ROC curves of the four methods to distinguish between known protein motifs and other DNA-binding protein motifs. Long-range contacts are assessed between a motif and promoters (enhancer-promoter contacts). e) Percent of dCTCF motif SNP that have a homologous interaction variable beta lower than the one of the dCTCF concensus motif. f) Comparison table of the methods.

We then compared GLMI with existing methods for their ability to identify genomic features known to be involved in long-range contacts. For this purpose, we compared GLMI with (1) enrichment test (ET) on highly confident chromatin interaction pairs as previously [26], (2) correlation (Cor) on highly confident chromatin interaction pairs [27] and (3) random forests (RF) discriminating highly confident chromatin interaction pairs from non-interacting pairs [28]. As a first and simple benchmark, we assessed the different methods to identify long-range contacts between protein binding sites of the same proteins (model (2)). We evaluated the ability to discriminate between architectural proteins known to be involved in long-range contacts (13 true positives including IBPs and cofactors) and random protein peaks (100 false positives) using receiver operating characteristic (ROC) curves. We observed that all four methods were very efficient to detect long-range contacts between known architectural protein binding sites (Fig 3a). In particular, GLMI and Cor showed perfect predictions (*AUC* = 1). RF and ET were also very accurate (*AUC* > 0.94). Previous benchmark was an easy task because it relied on random protein peaks whose binding was very different from real protein binding. For a more realistic benchmark, we then evaluated the ability to discriminate between motifs whose proteins are known to be involved in long-range contacts (7 true positives) and other DNA-binding protein motifs (83 false positives) using ROC curves. Using this benchmark, all the four methods performed less well (Fig 3b). However we found that GLMI clearly outperformed the three other methods to detect long-range contacts between DNA motifs known to be involved in chromatin interactions (*AUC*_*GLMI*_ = 0.855).

Another benchmark consisted in identifying long-range contacts between binding sites of a protein and active promoters. Here, as previously, we evaluated the ability to discriminate between architectural proteins known to be involved in enhancer-promoter contacts (13 true positives including IBPs and cofactors) and random protein peaks (100 false positives) using ROC curves. We observed that all four methods were very efficient to detect long-range contacts between known architectural protein binding sites and active promoters (Fig 3c). In particular, GLMI and Cor showed excellent predictions (*AUC*_*GLMI*_ = 0.985 and *AUC*_*Cor*_ = 1). We then evaluated the ability to discriminate between motifs whose proteins are known to be involved in enhancer-promoter contacts (7 true positives) and other DNA-binding protein motifs (83 false positives) using ROC curves. Both GLMI and Cor performed well (*AUC*_*GLMI*_ = 0.797 and *AUC*_*Cor*_ = 0.807; Fig 3d). Conversely, ET and RF showed lower perfomance (*AUC*_*ET*_ = 0.728 and *AUC*_*RF*_ = 0.601).

We next analyzed the impacts of mutations in the consensus dCTCF motif. Single nucleotide polymorphisms (SNPs) play an important role in common genetic diseases and recent works have uncovered differential long-range contacts due to variations in the CTCF motif in human [17, 29, 30]. Hence we evaluated the methods to detect the impacts of single nucleotide mutations in the dCTCF motif. For this purpose, we considered the dCTCF consensus motif AGGTGGCG (wild-type motif) [31] and generated dCTCF motifs with single nucleotide mutations for each position (mutated motifs). For instance, for the first position, the mutated motifs were TGGTGGCG, GGGTGGCG and CGGTGGCG. Over the 24 possible mutated motifs (8 positions × 3 alternative nucleotides), GLMI detected 17 motifs (71%; Fig 3e) with homologous interaction variable betas that were lower than the one of the wild-type motif, indicating that the corresponding mutations diminished the ability of dCTCF to bridge long-range contact. Compared to GLMI, other approaches showed lower performance (Cor: 14/24; RF = 10/24; ET = 8/24).

In addition to its better prediction performances, our model presents several theoretical advantages over the three other methods as summarized in Fig 3f. All the methods can assess long-range contacts between protein binding sites. However, GLMI is the only model that, at the same time, (1) accounts for the contact frequency which can vary among highly confident loops, (2) can deal with the presence of colocalization among proteins using conditional independence, (3) allows variable selection using lasso or stepwise, and (4) can assess the effect of cofactors by including higher-order interaction terms.

### Analysis of insulator binding protein motifs in *Drosophila*

**Fig 4:**
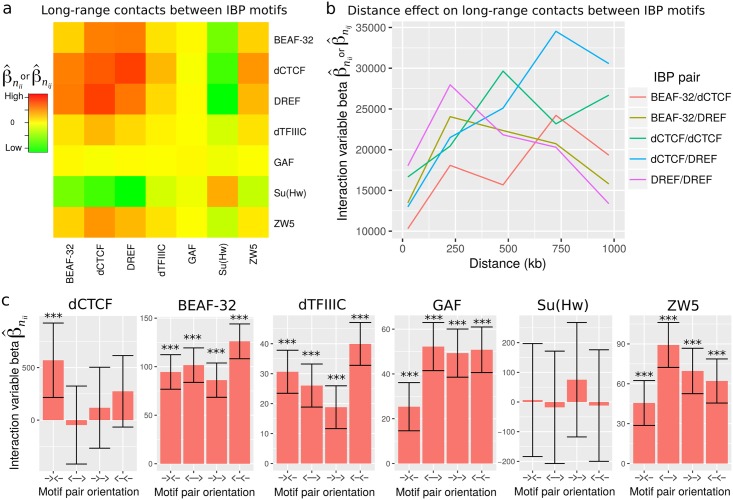
Analysis of long-range contacts between insulator binding protein (IBP) motifs. a) Long-range contacts between IBP motifs, as measured by interaction variable betas estimated using models (2) and (3). b) Long-range contacts between IBP motifs depending on the distance. c) Long-range contacts between IBP motifs depending on the motif pair orientation.

Given the biological validation of our model, we next sought to address the roles of IBP motifs in establishing or maintaining long-range interactions in *Drosophila*. We first assessed how IBP motifs were coupled to form loops (*i.e.* for all combinations of distant IBP motifs). For this purpose, we estimated homologous and heterologous interaction variable effects for any couple of IBP motifs using models (2) and (3), and using the same Hi-C data, distance range and resolution as above (Fig 4a). The strongest long-range contacts were between dCTCF and DREF motifs (${\hat{\beta }}_{n_{ij}}=2.8\times 10^{4}$, *p* < 10^−20^), between dCTCF motifs (${\hat{\beta }}_{n_{ii}}=2.4\times 10^{4}$, *p* < 10^−20^) and between DREF motifs (${\hat{\beta }}_{n_{ii}}=2\times 10^{4}$, *p* < 10^−20^). High levels of long-range contacts were also found between BEAF-32 and DREF motifs (${\hat{\beta }}_{n_{ij}}=1.9\times 10^{4}$, *p* < 10^−20^) and between BEAF32 and dCTCF motifs (${\hat{\beta }}_{n_{ij}}=1.9\times 10^{4}$, *p* < 10^−20^). Thus in *Drosophila*, chromatin loops not only involve dCTCF motifs but also DREF and BEAF-32 motifs that all work together. We then explored if these long-range contacts depended on the distance between motifs. At short distance (<100kb), long-range contacts were mainly detected between DREF motifs (${\hat{\beta }}_{n_{ii}}=1.8\times 10^{4}$, *p* < 10^−20^), whereas at long distance (> 750kb), they were more frequent between dCTCF and DREF motifs (${\hat{\beta }}_{n_{ij}}=3.5\times 10^{4}$, *p* = 7 × 10^−9^) (Fig 4b). In addition, long-range contacts between dCTCF motifs peaked at 500 kb. Our results therefore raise the possibility that long-range contacts between IBP motifs could be distant-dependent. This observation might provide a molecular explanation for the observed hierarchical nature of 3D chromatin structure [32, 33], for which loops could be formed at different scales by the interplay of specific proteins.

Next we sought to comprehensively test whether motif orientation could influence long-range contacts, as originally shown for CTCF motifs in human [10] and more generally in mammals [34]. We distinguished the motifs that were on the positive DNA strand (denoted +), from those that were on the negative DNA strand (denoted -). Then it was possible to compute four types of homologous interaction variables: **n**_*ii*+−_ = **z**_*iL*+_ × **z**_*iR*−_ (orientation →←), **n**_*ii*−+_ = **z**_*iL*−_ × **z**_*iR*+_ (orientation ←→), **n**_*ii*−−_ = **z**_*iL*−_ × **z**_*iR*−_ (orientation ←←), **n**_*ii*++_ = **z**_*iL*+_ × **z**_*iR*+_ (orientation →→). The corresponding models are detailed in Subsection Materials and Methods, The different models. Here we processed data at 1 kb resolution for better accuracy in distinguishing the different orientations. Similarly to in human and mammals, we found significant long-range contacts for motifs in convergent orientation (${\hat{\beta }}_{n_{ii}}=570$, *p* = 2 × 10^−3^), and no significant contacts for the 3 other possible orientations (←→, →→ and ←←; Fig 4c), revealing conservation of convergent CTCF mediated loops in agreement with 4C analyses [35]. We then assessed motif orientation for all other IBP motifs. Of note, the orientation of DREF TATCGATA motifs could not be assessed because of its palindromic property. For BEAF-32, dTFIIIC and Su(Hw) motifs, we could not detect any strong orientation effect (Fig 4c). Conversely, for GAF and ZW5 motifs, we found stronger contacts for motifs in divergent orientation (←→) compared to convergent orientation (→←), suggesting a different mode of binding of the corresponding protein to DNA or a different constraint depending of its interaction with cofactors. Thus motif orientation in loops depends on the protein involved, and the dependence on convergent orientation of motifs does not apply to all insulator binding proteins.

### Analysis of insulator binding protein sites in *Drosophila*

**Fig 5:**
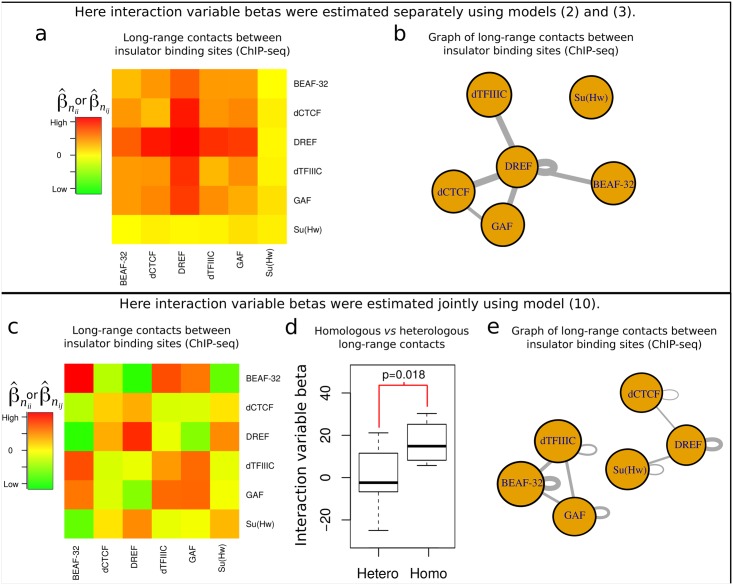
Analysis of long-range contacts between insulator binding protein (IBP) sites. a) Long-range contacts between IBP sites, as measured by interaction variable betas estimated separately (models (2) and (3)). b) Graph of long-range contacts (betas) between IBP sites estimated in a). c) Long-range contacts between insulator binding sites, as measured by interaction variable betas estimated jointly (model (10)). d) Comparison between homologous and heterologous interaction variable betas. e) Graph of long-range contacts (betas) between IBP sites estimated in c).

IBP binding sites might significantly vary depending on the cell type and stage. Hence we reanalyzed the roles of IBP binding in Kc167 *Drosophila* cells using available ChIP-seq data (same cell type with Hi-C data; ZW5 data were not available). As in the previous subsection, we estimated interaction effects for any couple of IBP motifs using models (2) and (3). Similarly to the analysis of IBP motifs, we observed high levels of long-range contacts involving DREF and dCTCF (Fig 5a). In particular, we found strong long-range contacts between distant DREF binding sites (${\hat{\beta }}_{n_{ii}}=147$, *p* < 10^−20^) and between dCTCF and DREF binding sites (${\hat{\beta }}_{n_{ij}}=133$, *p* < 10^−20^). However, we also observed strong long-range contacts between DREF and dTFIIIC (${\hat{\beta }}_{n_{ij}}=119$, *p* < 10^−20^), and between DREF and GAF (${\hat{\beta }}_{n_{ij}}=112$, *p* < 10^−20^), which could not be detected by previous analysis of IBP motifs. We then built a graph using estimated betas by adding an edge between two proteins *F*_*i*_ and *F*_*j*_ with a weight ${\hat{\beta }}_{n_{ij}}$, and by adding an edge between a protein *F*_*i*_ and itself with a weight ${\hat{\beta }}_{n_{ii}}$ (Fig 5b). Analysis of the graph clearly revealed the role of DREF as a hub, *i.e.* DREF was involved in many long-range contacts with other IBPs, such as BEAF-32, DREF, dTFIIIC and GAF. Such DREF-mediated loops might be in apparent contradiction with recent experiments showing that DREF motifs tag proximal activation of housekeeping genes, in contrast to long-range activation of developmental genes [36]. However such DREF-mediated loops can be explained by long-range contacts between promoters (${\hat{\beta }}_{n_{ii}}=203$, *p* < 10^−20^).

Previous results should be carrefully interpreted since IBPs often linearly colocalize (*i.e.* correlate) with each other on the chromosome [31]. Such correlations can lead to “indirect” long-range contacts between IBPs. For instance, if a loop is maintained by two distant dCTCF binding sites, and that BEAF-32 colocalizes to dCTCF, then it is likely that we will also observe loops between distant BEAF-32 and dCTCF sites, and even between BEAF-32 sites. The impact of such correlations between proteins in the study of 3D chromatin has been discussed in details [12]. Models (2) and (3) could not account for such correlations between IBPs because only one interaction variable term was included. Instead one should use another model that includes all possible interaction variable terms between IBPs (model (10), see Subsection Materials and methods, The different models). To better discard indirect long-range contacts between the 6 IBPs, we thus re-estimated interaction variable beta parameters using model (10) that included all marginal variables (6 variables, one for each IBP) and all interaction variables (21 variables, one for each combination of IBPs). Using model (10), we obtained rather different results (Fig 5c). We still observed strong long-range contacts between DREF binding sites (${\hat{\beta }}_{n_{ii}}=25$, *p* < 10^−11^). However other long-range contacts were observed such as between BEAF-32 sites (${\hat{\beta }}_{n_{ii}}=30$, *p* < 10^−20^). In turn, such analysis showed that an IBP tended to interact more with itself (homologous interactions) than with another IBP (heterologous interactions) (*p* = 0.018; Fig 5d), in agreement with insulator bodies observed by microscopy [37]. In addition, the model (10) allowed to infer negative and significant interaction effects, such as between distant DREF and BEAF-32 (${\hat{\beta }}_{n_{ij}}=-25$, *p* < 10^−11^), which could not be detected before. This negative effect means that BEAF-32 and DREF tend to avoid each other in long-range contacts, *i.e.* they tend to have a repulsive effect. This might reflect the known antagonistic relationship between BEAF-32 and DREF in competing for binding to overlapping binding sites [38, 39]. As previously, we built a graph of betas and could detect groups of IBPs that may cluster together through long-range contacts as found for the two connected components BEAF-32/dTFIIIC/GAF and DREF/Su(Hw)/dCTCF, respectively (Fig 5e). Interestingly, these two classes of IBPs that worked together in 3D were different from the two classes that were previously identified by 1D analysis: dCTCF/BEAF-32 and Su(Hw), respectively [40]. Such observations strenghtened the importance of analyzing protein complexes in 3D in complement to 1D analysis (see Discussion).

### Analysis of DNA-binding protein sites in human

In human and mammals, the main model of loop formation involves CTCF and cohesin [10, 17]. According to this model, a loop may form by the homodimerization of two CTCF proteins bound to two distant CTCF motifs that are in convergent orientation [10]. The loop also involves cohesin that is recruited by CTCF and that has the ability to entrap the two DNA fibers inside a ring. In addition to CTCF and cohesin, other architectural proteins have been recently uncovered such as ZNF143 [41] and PcG proteins [42]. In order to systematically analyze proteins mediating loops, we considered integrating available protein binding data (73 proteins) together with high-resolution Hi-C data in human GM12878 cells using our GLMI model. As previously done for *Drosophila*, we analyzed Hi-C data at 10 kb resolution and focused on 20kb-1Mb distances [10]. At this distance range, the Hi-C data comprised a very large number of bin pairs (around 22 millions), and hence, its analysis often required subsampling to few million pairs to achieve tractable regression parameter estimation. As for *Drosophila*, the log-log relation between Hi-C count and distance was linear at this distance range (*R*^2^ = 0.992, S2 Fig), supporting the use of the log-distance term in the model.

**Fig 6:**
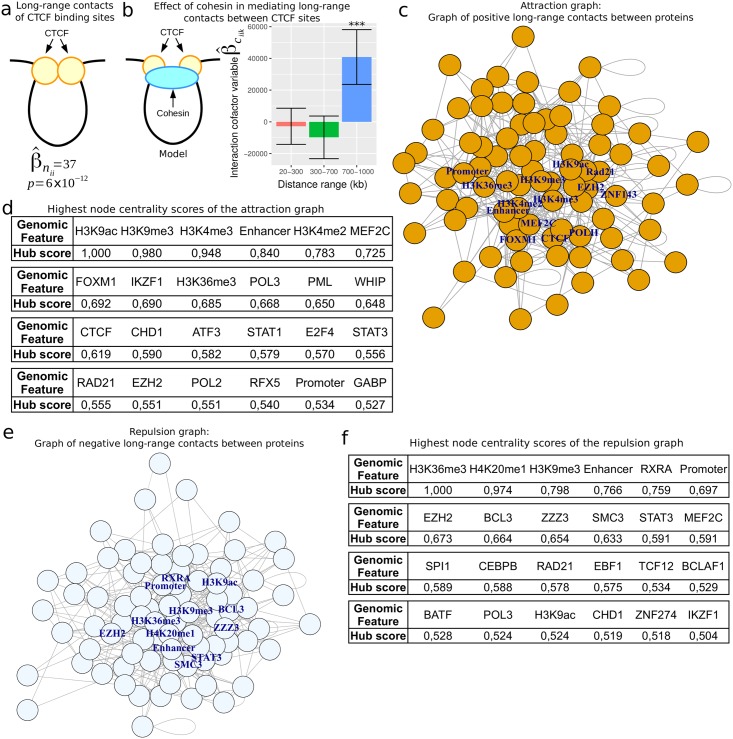
Analysis of long-range contacts between architectural protein binding (IBP) sites in human GM12878 cells. a) Long-range contacts between CTCF sites, and between Rad21 sites, as measured by interaction variable betas estimated using model (2). b) Effect of cohesin in mediating long-range contacts between CTCF sites. c) Attraction graph of long-range contacts between DNA-binding protein sites estimated using positive interaction variable betas from model (10). d) Highest node centrality scores from the attraction graph as measured by eigen decomposition. e) Repulsion graph of long-range contacts between DNA-binding protein sites estimated using negative interaction variable betas from model (10). f) Highest node centrality scores from the repulsion graph as measured by eigen decomposition.

We first investigated contacts between distant CTCF binding sites using model (2). As expected, we observed strong long-range contacts (${\hat{\beta }}_{n_{ii}}=37$, *p* = 6 × 10^−12^) [10]. Moreover high levels of long-range contacts were detected between cohesin subunit Rad21 binding sites as expected (${\hat{\beta }}_{n_{ii}}=89$, *p* < 10^−20^; Fig 6a) [10], as well as between cohesin subunit SMC3 (${\hat{\beta }}_{n_{ii}}=75$, *p* < 10^−20^). We then used the same approach to estimate long-range contacts for all 73 proteins available (S1 Table). Among the proteins that significantly interacted among themselves, we found several proteins known to colocalize to CTCF binding sites including YY1 (${\hat{\beta }}_{n_{ii}}=31$, *p* < 10^−20^), MAZ (${\hat{\beta }}_{n_{ii}}=16$, *p* < 10^−20^) and JUND (${\hat{\beta }}_{n_{ii}}=258$, *p* = 10^−9^) [7]. We also found P300, an important transcriptional coactivator [43] (${\hat{\beta }}_{n_{ii}}=264$, *p* < 10^−20^). In addition, histone marks including H3K27me3, H3K36me3, H3K4me2, H3K4me3, H3K9ac and H3K9me3 showed homologous long-range contacts, as previously shown by polymer simulations [44] (all ${\hat{\beta }}_{n_{ii}}>0.05$, *p* < 10^−20^). Curiously, H4K20me1 sites presented repulsive effects with each other (${\hat{\beta }}_{n_{ii}}=-0.07$, *p* < 10^−20^), indicating that distant H4K20me1 marked sites may avoid each other. We further estimated the well-known influence of cohesin in mediating long-range contacts between distant CTCF binding sites in human using model (4) [8, 10]. Interestingly, we found that the effect of cohesin depended on the distance between CTCF binding sites, with no significant contacts for short distances (20-300kb: ${\hat{\beta }}_{c_{iik}}=-3\times 10^{3}$, *p* = 0.63; 300-700kb: ${\hat{\beta }}_{c_{iik}}=-1\times 10^{4}$, *p* = 0.15) and significant contacts for long distances (700-1000kb: ${\hat{\beta }}_{c_{iik}}=4\times 10^{4}$, *p* = 3 × 10^−6^) (Fig 6b). This suggested that cohesin is required for stabilizing CTCF-mediated loops for long distances, but is not necessary for short distances for which homodimerization of CTCF might be sufficient. We also sought for other proteins whose loops could be mediated by cohesin for long distances (S2 Table). Most notably, we found that cohesin positively influences long-range contacts between architectural protein ZNF143 binding sites (${\hat{\beta }}_{c_{iik}}=4.8\times 10^{4}$, *p* = 2 × 10^−9^), between PolII binding sites (${\hat{\beta }}_{c_{iik}}=446$, *p* = 6 × 10^−16^), and between transcriptional factor binding sites (EGR1, ELF1, FOXM1, MAZ, MXI1, NRF1, YY1), which suggests a wider role for cohesin in mediating long-range contacts.

Further analyses of long-range contacts for every couple of proteins were performed using model (10) that included together all possible interaction variables. We considered 73 proteins, 7 histone modifications, active enhancers and active promoters. The model thus comprised (82 × 83)/2 = 3403 interaction variables. To deal with such a large number of interaction variables, we used a Poisson lasso estimation [45]. An interaction variable beta of zero was expected to reflect the absence of direct long-range contact between two proteins. From the estimated betas, we built a first graph that we called “attraction graph” by adding an edge between two proteins *F*_*i*_ and *F*_*j*_ if ${\hat{\beta }}_{n_{ij}}>0$, and by adding an edge between a protein *F*_*i*_ and itself if ${\hat{\beta }}_{n_{ii}}>0$ (Fig 6c). To identify hubs in the graph, we used eigenvector centrality that reflected how central is a node (Fig 6d). Both active and repressed chromatin marks as well as enhancers were the most central nodes (H3K9ac: score = 1; H3K9me3: score = 0.98; H3K4me3: score = 0.948; Enhancer: score = 0.84). Among DNA-binding proteins, CTCF and Rad21 showed high values (CTCF: score = 0.619; Rad21: score = 0.555). Surprisingly, however, other proteins MEF2C and FOXM1 presented the highest values (MEF2C: score = 0.725; FOXM1: score = 0.692). Previous studies showed that MEF2C is necessary for bone marrow B-lymphopoiesis (GM12878 is a lymphoblastoid cell line) [46], and that FOXM1 has an important role in maintenance of chromosomal segregation [47]. We then looked for cliques in the graph, *i.e.* a group of nodes that were all connected to each other (complete list in S3 Table). As expected, we found a clique composed of CTCF and the cohesin subunits Rad21 and SMC3, that are known to mediate together loops [10]. But we also found novel protein complexes that were specific to lymphocyte B such as the clique IKZF1/RFX5/PolII. IKZF1 plays a role in the development of lymphocytes [48], RFX5 is involved in bare lymphocyte syndrome [49] and polymerase II catalyzes gene transcription. In addition, we found many cliques involving Polymerase III (PolIII) such as the cliques MEF2C/RUNX3/PolIII and MEF2C/WHIP/PolIII, which might reflect the influence of architectural protein RNA polymerase III-associated factor (TFIIIC) at tRNA genes [2, 50].

Very little is known about repulsion effects between distant binding sites. Such repulsive effects could result from allosteric effects of loops [51], or factors that disassociate protein complexes involved in loops [52]. To investigate repulsive effects, we built a second graph that we called “repulsion graph” by adding an edge between two proteins *F*_*i*_ and *F*_*j*_ if ${\hat{\beta }}_{n_{ij}}<0$, and by adding an edge between a protein *F*_*i*_ and itself if ${\hat{\beta }}_{n_{ii}}<0$ (Fig 6e). The repulsion graph was very different from the attraction graph. Different histone marks were central in the repulsion graph, including H3K36me3 (score: 1) and H4K20me1 (score: 0.974), except histone mark H3K9me3 (score: 0.798) that was central in both the attraction and repulsion graphs (Fig 6f). Interestingly, we found that enhancers presented a high centrality score in the repulsion graph (score: 0.766), as found in the attraction graph. This result highlights the ability of enhancers to specifically interact with distant protein partner binding sites while avoiding others. Supporting this interpretation, we found enhancers to be in attraction with CFOS, NRF1 or POU2F2, and in repulsion with RXRA, NFE2 or P300. We then looked at pairs of proteins that were in repulsion. Most notably, we found CTCF to be in repulsion with EZH2, which might result from steric effects of CTCF-mediated loops [10] with Polycomb-mediated loops [42].

### The influence of DNA-binding proteins on enhancer-promoter interactions in human

**Fig 7:**
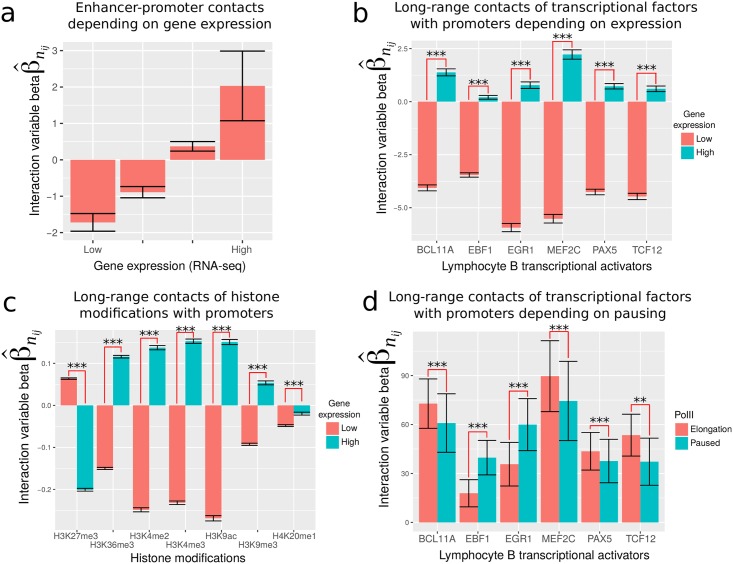
Influence of DNA-binding proteins and histone marks on enhancer-promoter contacts in human GM12878 cells. a) Enhancer-promoter contacts depending on gene expression, as measured by interaction variable betas estimated using model (3). b) Long-range contacts of transcriptional factors with promoters depending on gene expression. c) Long-range contacts of histone modifications with promoters depending on gene expression. d) Long-range contacts of transcriptional factors with promoters depending on PolII pausing or elongation.

Enhancer-promoter (EP) interactions play an essential role in the regulation of gene expression [14, 18]. Therefore, we explored the roles of DNA-binding proteins in establishing or maintaining EP interactions. Before assessing the role of proteins, we first measured long-range contacts between active enhancers and promoters depending on gene expression using model (3) (Fig 7a). We observed an attraction effect between active enhancers and highly expressed gene promoters (${\hat{\beta }}_{n_{ij}}=2$, *p* = 3 × 10^−5^), and conversely, a repulsion effect between active enhancers and low expressed gene promoters (${\hat{\beta }}_{n_{ij}}=-1.7$, *p* < 1 × 10^−20^), in complete agreement with the established positive influence of long-range contacts on gene expression [53]. To identify the influence of DNA-binding proteins, we then assessed the presence of long-range contacts between lymphocyte B transcriptional activator binding sites (ChIP-seq data) and promoters using the same model (3). All lymphocyte B transcriptional activators including BCL11A, EBF1, EGR1, MEF2C, PAX5 and TCF12 showed long-range contacts with highly expressed gene promoters, compared to weakly transcribed gene promoters (Fig 7b). This clearly showed that lymphocyte B transcriptional activators regulate expression of target genes through long-range contacts. Among the proteins available, we could not identify any that acted as silencers, *i.e.* proteins whose long-range contacts are high with low expressed gene promoters and low with highly expressed gene promoters. However when we focused on histone modifications, we found that long-range contacts of H3K27me3 mark were stronger to weakly transcribed gene promoters (${\hat{\beta }}_{n_{ij}}=0.06$, *p* < 10^−20^), compared to highly expressed gene promoters (${\hat{\beta }}_{n_{ij}}=-0.2$, *p* < 10^−20^) (Fig 7c). This suggested that H3K27me3 mark not only acts as a transcriptional silencer in linear proximity [54], but could also repress target genes at distance through loops. Conversely, active marks such as H3K4me3 and H3K9ac interacted more with highly expressed genes. Because enhancer-promoter contacts were previously shown to be associated with Polymerase II pausing [18], we then assessed enhancer-promoter interactions depending on gene transcription pausing. As expected, we found higher EP contacts at paused genes (${\hat{\beta }}_{n_{ij}}=62.2$, *p* = 10^−3^), compared to genes in elongation (${\hat{\beta }}_{n_{ij}}=49.3$, *p* = 2 × 10^−3^). We then looked at the influence of DNA-binding proteins (Fig 7d). For instance, EBF1 sites showed higher long-range contacts with promoters of genes in pause (${\hat{\beta }}_{n_{ij}}=39.7$, *p* = 1 × 10^−13^), compared to those in elongation (${\hat{\beta }}_{n_{ij}}=17.8$, *p* = 3 × 10^−5^), in agreement with [18]. But, surprisingly, we also found that BCL11A sites showed higher long-range contacts with promoters of genes in elongation (${\hat{\beta }}_{n_{ij}}=72.8$, *p* < 10^−20^) than with genes in pause (${\hat{\beta }}_{n_{ij}}=60.9$, *p* = 2 × 10^−11^). These observations suggest that, depending on the protein involved, long-range contacts with promoters are not always associated with pausing, but could also be linked to elongation.

### Conclusion

Here, we propose to use a generalized linear regression with interactions (GLMI) to study the roles of genomic features such as DNA-binding proteins, motifs or promoters to bridge long-range contacts in the genome, depending on transcriptional status or motif orientation. GLMI has multiple assets over existing approaches such as enrichment test, correlation and random forests. Compared to enrichment test [2, 55] or correlation [27] that respectively assesses the protein enrichment or correlation at highly confident loops, GLMI quantitatively links the frequency of all long-range contacts to complex co-occupancies of proteins while accounting for known Hi-C biases and polymer background. Moreover, GLMI accounts for colocalizations among protein binding, a strong issue when analyzing protein binding sites known to largely overlap over the genome. In contrast to random forests [28] which are efficient predictive models but sometimes poor explanatory ones, GLMI allows to identify key chromatin loop driver proteins and motifs. GLMI can also uncover numerous mechanisms behind loop formation using higher-order interaction terms and proper confounding variables. For instance, GLMI can determine if a cofactor is necessary to mediate long-range contacts between distant protein binding sites.

Using real *Drosophila* Hi-C and ChIP-seq data, we validate numerous GLMI predictions of long-range contacts that involve insulator binding proteins, cofactors and motifs, and which were confirmed by previous microscopy and mutational studies. For instance, our model estimates long-range contacts between distant BEAF-32 motifs, which were previously observed with both fluorescence cross-correlation spectroscopy [22] and high-resolution microscopy [23]. In addition, our model finds a mediating role of CP190 in bridging long-range contacts between distant BEAF-32 and GAF binding sites, in agreement with mutational experiments [19]. Of interest, GLMI analyses highlight a role of cohesin in stabilizing long-range contacts between CTCF sites in *Drosophila*, similarly to its role in human [7]. Supporting this role, we show that such influence is reduced upon cohesin subunit Rad21 depletion. It has to be noted that the absence of complete loss of contacts between CTCF sites after Rad21 depletion can be explained by the fast turnover of chromosome-bound cohesin in interphase [56]. Moreover, GLMI outperforms enrichment test, correlation and random forests in the identification of known architectural proteins and motifs, and in the detection of the effects of mutations in the dCTCF motif.

The proposed model also uncovers several novel results. In *Drosophila*, GAF and ZW5 motifs are shown to act in divergent orientation to form loops, in contrast to CTCF motifs that are found in convergent orientation in *Drosophila* and human [10, 17], suggesting a different mode of action of corresponding proteins. In addition, we identify two groups of proteins that act in 3D to form loops. The first group comprises BEAF-32, dTFIIIC and GAF, and the other group includes DREF, Su(Hw) and dCTCF. Those groups are different from the ones observed with 1D analysis only (*i.e.* linear colocalization on the genome) [40], highlighting the importance of 3D analysis using GLMI. In human, we identify numerous long-range contacts between protein binding sites. In addition to the well-known protein complex CTCF/RAD21/SMC3, we uncover new protein complexes that are specific to lymphocyte B such as IKZF1/RFX5. We also found that enhancers could be either in long-range contact or repulsion with certain protein binding sites, highlighting potential specificity in selecting protein partners for long-range contacts. Our observations therefore support the idea that enhancer-promoter contacts are not solely driven by insulators or TAD borders that physically constrain such long-range interactions [29, 36, 57]. Rather, enhancer-promoter contacts may also be encoded by the specificity of protein-protein interactions. In addition, our results suggest that repressive mark H3K27me3 does not only repress genes that are contigous [54], but it could also repress from a distance through the juxtaposition of H3K27me3 with genes in 3D. We also find that, depending on the protein involved, long-range enhancer-promoter contacts are not always favored by PolII pausing [18], which may highlight distinct mechanisms by which proteins can influence transcription-associated long-range contacts.

There are several limitations of the proposed approach. First, the present analysis is restricted to a 10-kb resolution because of the quadratic complexity of Hi-C data. Second, our analysis is limited by the amount of higher-order interaction variable parameters that can be learned within the same model (full model) using current parameter learning programs. Most notably, all possible interaction cofactor variables cannot be included in the same model because of the cubic complexity of such model, and hence they are learned separately instead (using models (4) and (5)). In addition, although generalized linear models can include interactions of any order involving large protein complexes (for instance, complexes of more than 4 proteins), parameter learning is limited by the availability of data and computational resources. Increasing depth of Hi-C data will allow inference of more complex models in the near future. Moreover the development of new big data learning algorithms could be used to process the data at a higher resolution that would allow in-depth analysis of 3D chromatin drivers [58]. An alternative to the exploration of all possible higher-order interactions together might be to guide the search using prior information, such as protein-protein interaction network [55]. Lastly, in order to explore all possible higher-order interaction variables within the same model (full model), one should use a lasso regression model with hierarchically constrained interactions [59].

## Materials and methods

### Hi-C data

We used publicly available high-throughput chromatin conformation capture (Hi-C) data from Gene Expression Omnibus (GEO) accession GSE62904 [21]. Hi-C experiments have been done for *Drosophila melanogaster* wild-type and Rad21 knock-down Kc167 cells with DpnII restriction enzyme. Hi-C data were binned at 1 and 10 kb resolutions.

For human data analysis, we used publicly available Hi-C data of lymphoblastoid cells GM12878 cells from Gene Expression Omnibus (GEO) accession GSE63525 [10]. We used Hi-C data binned at 10 kb resolution.

### ChIP-seq data

For *Drosophila* analysis, we used publicly available binding profiles of chromatin proteins of *Drosophila melanogaster* wild-type embryonic Kc167 cells. ChIP-seq data for CP190, Su(Hw), dCTCF and BEAF-32 were obtained from GEO accession GSE30740 [60]. ChIP-seq data for Barren (condensin I), Cap-H2 (condensin II), Chromator, Rad21 (cohesin), GAF and dTFIIIC were obtained from GEO accession GSE54529 [9]. ChIP-seq data for DREF and L(3)Mbt were obtainted from GEO accession GSE62904 [21]. ChIP-seq data for Fs(1)h-L and Fs(1)h-LS were obtained from GEO accession GSE42086 [25]. Peak calling was done using MACS 2.1.0 (https://github.com/taoliu/MACS).

For human analysis, we used publicly available binding peaks of 73 chromatin proteins (RAD21, CTCF, YY1, ZBTB33, MAZ, JUND, ZNF143, EZH2, ATF2, ATF3, BATF, BCL11A, BCL3, BCLAF1, BHLHE40, BRCA1, CEBPB, CFOS, CHD1, CHD2, CMYC, COREST, E2F4, EBF1, EGR1, ELF1, ELK1, FOXM1, GABP, IKZF1, IRF4, MAX, MEF2C, MTA3, MXI1, NFATC1, NFE2, NFIC, NFKB, NFYA, NFYB, NRF1, NRSF, P300, PAX5, PBX3, PML, POL2, POL3, POU2F2, RFX5, RUNX3, RXRA, SIN3A, SIX5, SMC3, SP1, SPI1, SRF, STAT1, STAT3, STAT5, TBLR1, TBP, TCF12, TCF3, TR4, USF1, USF2, WHIP, ZEB1, ZNF274, ZZZ3) and histone marks (H3K27me3, H3K36me3, H3K4me2, H3K4me3, H3K9ac, H3K9me3, H4K20me1) of GM12878 cells from ENCODE [61]. We downloaded peaks that were uniformly processed (Uniform Peaks).

### Functional elements

For human analysis, we divided promoters into quartiles of gene expression using RNA-seq data [61]. We also divided promoters into quartiles of gene pausing and into quartiles of gene elongation using PolII ChIP-seq data [61]. For enhancer mapping, we used lymphocyte of B lineage differentially expressed enhancers identified from the Fantom5 project [62].

### DNA motifs

For both *Drosophila* and human analyses, we used transcription factor binding site (TFBS) motifs from the MotifMap database (http://motifmap.ics.uci.edu/).

### Power-law distribution testing

The proposed GLMI assumed a linear relation between logarithm of Hi-C counts and the logarithm of distance between bins as previously shown in [5]. This assumption only holds locally, *i.e.* for a specific distance scale. Hence we restricted GLM modeling to a certain range of distances, *e.g.* for 20kb to 1Mb. In addition, we tested this assumption on data before using GLMI. We considered that this assumption holds when the *R*^2^ > 0.95.

### Occupancy variables z

Before computing variables for the GLMI presented above, intermediate variables from the genomic features such as DNA-binding proteins needed to be calculated. Intermediate “occupancy” variable **z**_*i*_ denoted the presence (**z**_*i*_ = 1) or absence (**z**_*i*_ = 0) of the protein *F*_*i*_ within the genomic bin. If the protein only overlapped 60% of the genomic bin, then **z**_*i*_ = 0.6.

### The different models

Here are described the different models derived from model (1) that we used. In order to assess a homologous interaction variable **n**_*ii*_ = **z**_*iL*_ × **z**_*iR*_ (here **g** = **n**_*ii*_), model (1) becomes:

$$\begin{array}{lll} \log\left(E\left[y|X\right]\right) & = & \beta_{0}+\beta_{d}d+\beta_{B}B+\beta_{C}C+\beta_{g}g\\ & = & \beta_{0}+\beta_{d}d+\beta_{B}B+\beta_{m_{i}}m_{i}+\beta_{n_{ii}}n_{ii} \end{array}$$ (2)

Following the hierarchy principle in (generalized) linear models, the assessment of a statistical interaction variable, such as **n**_*ii*_ = **z**_*iL*_ × **z**_*iR*_, must include both **z**_*iL*_ and **z**_*iR*_ as confounding variables. Because **z**_*iL*_ and **z**_*iR*_ are identically associated to **y** (the attribution for left and right bins is arbitrary), their values are averaged to give $m_{i}=\frac{1}{2}\left(z_{iL}+z_{iR}\right)$. Hence **C** = **m**_*i*_ is used as a confounder of **n**_*ii*_.

In order to assess a heterologous interaction variable $n_{ij}=\frac{1}{2}\left(z_{iL}\times z_{jR}+z_{jL}\times z_{iR}\right)$ (here **g** = **n**_*ij*_), model (1) becomes:

$$\begin{array}{lll} \log\left(E\left[y|X\right]\right) & = & \beta_{0}+\beta_{d}d+\beta_{B}B+\beta_{C}C+\beta_{g}g\\ & = & \beta_{0}+\beta_{d}d+\beta_{B}B+\beta_{m_{i}}m_{i}+\beta_{m_{j}}m_{j}+\beta_{n_{ij}}n_{ij} \end{array}$$ (3)

Following the hierarchy principle, **z**_*iL*_, **z**_*iR*_, **z**_*jL*_ and **z**_*jR*_ have to be included as confounding variables. As previously, **z**_*iL*_ and **z**_*iR*_ are averaged to give $m_{i}=\frac{1}{2}\left(z_{iL}+z_{iR}\right)$. Similarly, **z**_*jL*_ and **z**_*jR*_ are averaged to give $m_{j}=\frac{1}{2}\left(z_{jL}+z_{jR}\right)$. Hence **C** = {**m**_*i*_, **m**_*j*_} is used as confounder of **n**_*ij*_.

In order to assess a homologous interaction cofactor variable **c**_*iik*_ = **n**_*ii*_ × **n**_*kk*_ (here **g** = **c**_*iik*_), model (1) becomes:

$$\begin{array}{lll} \text{log}\left(\text{E}\left[y|X\right]\right) & = & \beta_{0}+\beta_{d}d+\beta_{B}B+\beta_{C}C+\beta_{g}g\\ & = & \beta_{0}+\beta_{d}d+\beta_{B}B+\beta_{m_{i}}m_{i}+\beta_{m_{k}}m_{k}+\beta_{m_{ik}}m_{ik}+\beta_{n_{ii}}n_{ii}+\beta_{n_{kk}}n_{kk}+\beta_{n_{ik}}n_{ik}\\ & + & \beta_{n_{ii}\times m_{k}}\left(n_{ii}\times m_{k}\right)+\beta_{n_{kk}\times m_{i}}\left(n_{kk}\times m_{i}\right)+\beta_{c_{iik}}c_{iik}, \end{array}$$ (4)

Here variable **c**_*iik*_ is a four-way interaction term and hence there are a large number of confounding variables included in variable set **C** = {**m**_*i*_, **m**_*k*_, **m**_*ik*_, **n**_*ii*_, **n**_*kk*_, **n**_*ik*_, **n**_*ii*_ × **m**_*k*_, **n**_*kk*_ × **m**_*i*_}. We need to introduce a new type of variable, noted **m**_*ij*_, the average of product **z**_*iL*_ × **z**_*jL*_ and product **z**_*iR*_ × **z**_*jR*_ ($m_{ij}=\frac{1}{2}\left(z_{iL}\times z_{jL}+z_{iR}\times z_{jR}\right)$). For a detailed explanation of the confounder set **C**, see S1 Appendix, Confounder sets.

In order to assess a heterologous interaction cofactor variable **c**_*ijk*_ = **n**_*ij*_ × **n**_*kk*_ (here **g** = **c**_*ijk*_), model (1) becomes:

$$\begin{array}{lll} \text{log}\left(\text{E}\left[y|X\right]\right) & = & \beta_{0}+\beta_{d}d+\beta_{B}B+\beta_{C}C+\beta_{g}g\\ & = & \beta_{0}+\beta_{d}d+\beta_{B}B+\beta_{m_{i}}m_{i}+\beta_{m_{j}}m_{j}+\beta_{m_{k}}m_{k}+\beta_{m_{ik}}m_{ik}+\beta_{m_{jk}}m_{jk}\\ & + & \beta_{n_{ij}}n_{ij}+\beta_{n_{jk}}n_{jk}+\beta_{n_{ik}}n_{ik}+\beta_{n_{kk}}n_{kk}\\ & + & \beta_{n_{ij}\times m_{k}}n_{ij}\times m_{k}+\beta_{n_{kk}\times m_{i}}n_{kk}\times m_{i}+\beta_{n_{kk}\times m_{j}}n_{kk}\times m_{j}+\beta_{c_{ijk}}c_{ijk}. \end{array}$$ (5)

Here variable **c**_*ijk*_ is a four-way interaction term and hence there are a large number of confounding variables included in variable set **C** = {**m**_*i*_, **m**_*j*_, **m**_*k*_, **m**_*ik*_, **m**_*jk*_, **n**_*ij*_, **n**_*jk*_, **n**_*ik*_, **n**_*kk*_, **n**_*ij*_ × **m**_*k*_, **n**_*kk*_ × **m**_*i*_, **n**_*kk*_ × **m**_*j*_}. For a detailed explanation of the confounder set **C**, see S1 Appendix, Confounder sets.

In addition, we formulated models for homologous interaction variables, depending on motif pair orientation. For a pair of motifs in convergent orientation (→←), model (1) becomes:

$$\begin{array}{lll} \text{log}\left(\text{E}\left[y|X\right]\right) & = & \beta_{0}+\beta_{d}d+\beta_{B}B+\beta_{C}C+\beta_{g}g\\ & = & \beta_{0}+\beta_{d}d+\beta_{B}B+\beta_{z_{iL+}}z_{iL+}+\beta_{z_{iR-}}z_{iR-}+\beta_{n_{ii+-}}n_{ii+-} \end{array}$$ (6)

with **n**_*ii*+−_ = **z**_*iL*+_ × **z**_*iR*−_. Symbol “+” denoted motifs that were on the forward DNA strand, while symbol “-” denoted motifs that were on the reverse DNA strand. For instance, variable **z**_*iL*+_ was the occupancy of a motif on the forward DNA strand within genomic bins.

For a pair of motifs in divergent orientation (←→), model (1) becomes:

$$\begin{array}{lll} \text{log}\left(\text{E}\left[y|X\right]\right) & = & \beta_{0}+\beta_{d}d+\beta_{B}B+\beta_{C}C+\beta_{g}g\\ & = & \beta_{0}+\beta_{d}d+\beta_{B}B+\beta_{z_{iL-}}z_{iL-}+\beta_{z_{iR+}}z_{iR+}+\beta_{n_{ii-+}}n_{ii-+}, \end{array}$$ (7)

with **n**_*ii*−+_ = **z**_*iL*−_ × **z**_*iR*+_.

For a pair of motifs in same orientation (→→), model (1) becomes:

$$\begin{array}{lll} \text{log}\left(\text{E}\left[y|X\right]\right) & = & \beta_{0}+\beta_{d}d+\beta_{B}B+\beta_{C}C+\beta_{g}g\\ & = & \beta_{0}+\beta_{d}d+\beta_{B}B+\beta_{z_{iL+}}z_{iL+}+\beta_{z_{iR+}}z_{iR+}+\beta_{n_{ii++}}n_{ii++}, \end{array}$$ (8)

with **n**_*ii*++_ = **z**_*iL*+_ × **z**_*iR*+_.

For a pair of motifs in same orientation (←←), model (1) becomes:

$$\begin{array}{lll} \text{log}\left(\text{E}\left[y|X\right]\right) & = & \beta_{0}+\beta_{d}d+\beta_{B}B+\beta_{C}C+\beta_{g}g\\ & = & \beta_{0}+\beta_{d}d+\beta_{B}B+\beta_{z_{iL-}}z_{iL-}+\beta_{z_{iR-}}z_{iR-}+\beta_{n_{ii--}}n_{ii--}, \end{array}$$ (9)

with **n**_*ii*−−_ = **z**_*iL*−_ × **z**_*iR*−_.

Moreover, we formulated an additional “full” model where all possible homologous and heterologous interaction variables were included. For instance, if we study two proteins *F*_*i*_ and *F*_*j*_ that tend to linearly colocalize, then the following “full” model would be:

$$\begin{array}{lll} \text{log}\left(\text{E}\left[y|X\right]\right) & = & \beta_{0}+\beta_{d}d+\beta_{B}B+\beta_{C}C+\beta_{G}G,\\ & = & \beta_{0}+\beta_{d}d+\beta_{B}B+\beta_{m_{i}}m_{i}+\beta_{m_{j}}m_{j}+\beta_{n_{ii}}n_{ii}+\beta_{n_{jj}}n_{jj}+\beta_{n_{ij}}n_{ij}, \end{array}$$ (10)

where **G** is the set of all possible homologous and heterologous interaction variables. Here **G** = {**n**_*ii*_, **n**_*jj*_, **n**_*ij*_} for two proteins *F*_*i*_ and *F*_*j*_. The confounder set **C** = {**m**_*i*_, **m**_*j*_} includes all marginal variables.

### Implementation

The general linear regression with interactions is implemented in R language. The model is available in the R package “HiCglmi” which can be downloaded from the Comprehensive R Archive Network.

## Supporting information

S1 Appendix Bias variable computation and confounder sets. (PDF)

S1 Fig Log-log relation between Hi-C count and distance between bins in *Drosophila*. 20 kb resolution for distances comprised between 10kb and 1Mb. *Drosophila* Kc167 cell data.
(PDF)

S2 Fig Log-log relation between Hi-C count and distance between bins in human. 20 kb resolution for distances comprised between 10kb and 1Mb. Human GM12878 cell data.
(PDF)

S1 Table Long-range contacts between same genomic feature. Long-range contacts measured by homologous interaction variable betas. GM12878 cell ChIP-seq data.
(PDF)

S2 Table Mediating effect of cohesin (Rad21 subunit) on long-range contacts between same genomic feature. Mediating effect of cohesin measured by homologous interaction cofactor variable betas. GM12878 cell ChIP-seq data.
(PDF)

S3 Table Cliques from the attraction graph. GM12878 cell ChIP-seq data.
(PDF)
