# Supplementary material for: Uncovering direct and indirect molecular determinants of chromatin loops using a computational integrative approach
Source: PLoS Comput Biol. 2017 May 23;13(5):e1005538. doi: 10.1371/journal.pcbi.1005538 (PMC5462476; doi:10.1371/journal.pcbi.1005538)
Supplement: S1 Appendix — (PDF) [file pcbi.1005538.s001.pdf]

S1 Appendix. Bias variable computation and confounder sets.

Bias variable computation

We computed bias variables $\mathbf{B} = \{\mathbf{len}, \mathbf{GC}, \mathbf{map}\}$ including fragment length (**len**), GC-content (**GC**) and mappability (**map**). First, we truncated each fragment end up to 500 bp, and calculated the total length of truncated fragment ends in a bin b (len_b). We further calculated the GC content within a 200 bp region upstream of each fragment end (GC content feature) and the mappability score within a 500 bp region next to each fragment end (mappability feature). GC content and mappability are defined as the average of the corresponding features among all fragment ends falling into the bin (GC_b and map_b, resp.). For a pair of bins L and R, variable **len** is calculated as $\log(len_L \times len_R)$. Similarly, **GC** and **map** are calculated as $\log(GC_L \times GC_R)$ and as $\log(map_L \times map_R)$, resp. We used HiTC R package to compute bias variables (https://www.bioconductor.org/packages/release/bioc/html/HiTC.html).

Confounder sets

Here are the detailed explanations of the confounder sets $\mathbf{C}$ used in models (4-5) presented in the main text.

Model (4) consists in assessing a "homologous interaction cofactor" variable $\mathbf{c}_{iik} = \mathbf{n}_{ii} \times \mathbf{n}_{kk} = \mathbf{z}_{iL} \times \mathbf{z}_{iR} \times \mathbf{z}_{kL} \times \mathbf{z}_{kR}$. Following the hierarchy principle, variable $\mathbf{c}_{iik}$ is confounded by the set $\mathbf{C}$ defined as follows:

$$\begin{aligned}\mathbf{C} = \{&\mathbf{z}_{iL}, \mathbf{z}_{iR}, \mathbf{z}_{kL}, \mathbf{z}_{kR},\\ &\mathbf{z}_{iL} \times \mathbf{z}_{iR}, \mathbf{z}_{kL} \times \mathbf{z}_{kR}, \mathbf{z}_{iL} \times \mathbf{z}_{kR}, \mathbf{z}_{iR} \times \mathbf{z}_{kL}, \mathbf{z}_{iL} \times \mathbf{z}_{kL}, \mathbf{z}_{iR} \times \mathbf{z}_{kR},\\ &\mathbf{z}_{iL} \times \mathbf{z}_{iR} \times \mathbf{z}_{kL}, \mathbf{z}_{iL} \times \mathbf{z}_{iR} \times \mathbf{z}_{kR}, \mathbf{z}_{kL} \times \mathbf{z}_{kR} \times \mathbf{z}_{iL}, \mathbf{z}_{kL} \times \mathbf{z}_{kR} \times \mathbf{z}_{iR}\}\end{aligned}$$

As previously, variables $\mathbf{z}_{iL}$ and $\mathbf{z}_{iR}$ are averaged to give $\frac{1}{2}(\mathbf{z}_{iL} + \mathbf{z}_{iR}) = \mathbf{m}_i$. Similarly, variables $\mathbf{z}_{kL}$ and $\mathbf{z}_{kR}$ are averaged to give $\frac{1}{2}(\mathbf{z}_{kL} + \mathbf{z}_{kR}) = \mathbf{m}_k$. Variable $\mathbf{z}_{iL} \times \mathbf{z}_{iR}$ is simply $\mathbf{n}_{ii}$. Variable $\mathbf{z}_{kL} \times \mathbf{z}_{kR}$ is simply $\mathbf{n}_{kk}$. Variables $\mathbf{z}_{iL} \times \mathbf{z}_{kR}$ and $\mathbf{z}_{iR} \times \mathbf{z}_{kL}$ are identically associated to $\mathbf{y}$, and thus they are averaged to give $\mathbf{n}_{ik} = \frac{1}{2}(\mathbf{z}_{iL} \times \mathbf{z}_{kR} + \mathbf{z}_{kL} \times \mathbf{z}_{iR})$. Variables $\mathbf{z}_{iL} \times \mathbf{z}_{kL}$ and $\mathbf{z}_{iR} \times \mathbf{z}_{kR}$ are identically associated to $\mathbf{y}$, and thus they are averaged to give $\mathbf{m}_{ik} = \frac{1}{2}(\mathbf{z}_{iL} \times \mathbf{z}_{kL} + \mathbf{z}_{iR} \times \mathbf{z}_{kR})$. Variables $\mathbf{z}_{iL} \times \mathbf{z}_{iR} \times \mathbf{z}_{kL}$ and $\mathbf{z}_{iL} \times \mathbf{z}_{iR} \times \mathbf{z}_{kR}$ are identically associated to $\mathbf{y}$, and thus they are averaged to

give $\frac{1}{2}(\mathbf{z}_{iL} \times \mathbf{z}_{iR} \times \mathbf{z}_{kL} + \mathbf{z}_{iL} \times \mathbf{z}_{iR} \times \mathbf{z}_{kR}) = \frac{1}{2}(\mathbf{z}_{iL} \times \mathbf{z}_{iR} \times (\mathbf{z}_{kL} + \mathbf{z}_{kR})) = (\mathbf{z}_{iL} \times \mathbf{z}_{iR}) \times \frac{1}{2}(\mathbf{z}_{kL} + \mathbf{z}_{kR}) = \mathbf{n}_{ii} \times \mathbf{m}_k$. Variables $\mathbf{z}_{kL} \times \mathbf{z}_{kR} \times \mathbf{z}_{iL}$ and $\mathbf{z}_{kL} \times \mathbf{z}_{kR} \times \mathbf{z}_{iR}$ are identically associated to $\mathbf{y}$, and thus they are averaged to give $\frac{1}{2}(\mathbf{z}_{kL} \times \mathbf{z}_{kR} \times \mathbf{z}_{iL} + \mathbf{z}_{kL} \times \mathbf{z}_{kR} \times \mathbf{z}_{iR}) = \frac{1}{2}(\mathbf{z}_{kL} \times \mathbf{z}_{kR} \times (\mathbf{z}_{iL} + \mathbf{z}_{iR})) = (\mathbf{z}_{kL} \times \mathbf{z}_{kR}) \times \frac{1}{2}(\mathbf{z}_{iL} + \mathbf{z}_{iR}) = \mathbf{n}_{kk} \times \mathbf{m}_i$. These simplifications give a new set $\mathbf{C} = \{\mathbf{m}_i, \mathbf{m}_k, \mathbf{m}_{ik}, \mathbf{n}_{ii}, \mathbf{n}_{kk}, \mathbf{n}_{ik}, \mathbf{n}_{ii} \times \mathbf{m}_k, \mathbf{n}_{kk} \times \mathbf{m}_i\}$ as confounder of $\mathbf{c}_{iik}$.

Model (5) consists in assessing a "heterologous interaction cofactor" variable $\mathbf{c}_{ijk} = \mathbf{n}_{ij} \times \mathbf{n}_{kk} = \frac{1}{2}(\mathbf{z}_{iL} \times \mathbf{z}_{jR} \times \mathbf{z}_{kL} \times \mathbf{z}_{kR} + \mathbf{z}_{jL} \times \mathbf{z}_{iR} \times \mathbf{z}_{kL} \times \mathbf{z}_{kR})$. Following the hierarchy principle, variable $\mathbf{c}_{ijk}$ is confounded by the set $\mathbf{C}$ defined as follows:

$$\begin{aligned}\mathbf{C} = \{&\mathbf{z}_{iL}, \mathbf{z}_{iR}, \mathbf{z}_{jL}, \mathbf{z}_{jR}, \mathbf{z}_{kL}, \mathbf{z}_{kR},\\ &\mathbf{z}_{iL} \times \mathbf{z}_{jR}, \mathbf{z}_{jL} \times \mathbf{z}_{iR}, \mathbf{z}_{iL} \times \mathbf{z}_{kR}, \mathbf{z}_{kL} \times \mathbf{z}_{iR}, \mathbf{z}_{jL} \times \mathbf{z}_{kR}, \mathbf{z}_{jR} \times \mathbf{z}_{kL},\\ &\mathbf{z}_{kL} \times \mathbf{z}_{kR}, \mathbf{z}_{iL} \times \mathbf{z}_{kL}, \mathbf{z}_{kR} \times \mathbf{z}_{iR}, \mathbf{z}_{jL} \times \mathbf{z}_{kL}, \mathbf{z}_{kR} \times \mathbf{z}_{jR},\\ &\mathbf{z}_{iL} \times \mathbf{z}_{jR} \times \mathbf{z}_{kL}, \mathbf{z}_{iL} \times \mathbf{z}_{jR} \times \mathbf{z}_{kR}, \mathbf{z}_{iR} \times \mathbf{z}_{kL} \times \mathbf{z}_{kR}, \mathbf{z}_{iL} \times \mathbf{z}_{kL} \times \mathbf{z}_{kR},\\ &\mathbf{z}_{jR} \times \mathbf{z}_{kL} \times \mathbf{z}_{kR}, \mathbf{z}_{jL} \times \mathbf{z}_{kL} \times \mathbf{z}_{kR}, \mathbf{z}_{jL} \times \mathbf{z}_{iR} \times \mathbf{z}_{kL}, \mathbf{z}_{jL} \times \mathbf{z}_{iR} \times \mathbf{z}_{kR}\}\end{aligned}$$

As previously, variables $\mathbf{z}_{iL}$ and $\mathbf{z}_{iR}$ are identically associated to $\mathbf{y}$, and thus they are averaged to give $\frac{1}{2}(\mathbf{z}_{iL} + \mathbf{z}_{iR}) = \mathbf{m}_i$. Variables $\mathbf{z}_{jL}$ and $\mathbf{z}_{jR}$ are identically associated to $\mathbf{y}$, and thus they are averaged to give $\frac{1}{2}(\mathbf{z}_{jL} + \mathbf{z}_{jR}) = \mathbf{m}_j$. Variables $\mathbf{z}_{kL}$ and $\mathbf{z}_{kR}$ are identically associated to $\mathbf{y}$, and thus they are averaged to give $\frac{1}{2}(\mathbf{z}_{kL} + \mathbf{z}_{kR}) = \mathbf{m}_k$. Variables $\mathbf{z}_{iL} \times \mathbf{z}_{jR}$ and $\mathbf{z}_{iR} \times \mathbf{z}_{jL}$ are identically associated to $\mathbf{y}$, and thus they are averaged to give $\mathbf{n}_{ij} = \frac{1}{2}(\mathbf{z}_{iL} \times \mathbf{z}_{jR} + \mathbf{z}_{jL} \times \mathbf{z}_{iR})$. Variables $\mathbf{z}_{iL} \times \mathbf{z}_{kR}$ and $\mathbf{z}_{iR} \times \mathbf{z}_{kL}$ are identically associated to $\mathbf{y}$, and thus they are averaged to give $\mathbf{n}_{ik} = \frac{1}{2}(\mathbf{z}_{iL} \times \mathbf{z}_{kR} + \mathbf{z}_{kL} \times \mathbf{z}_{iR})$. Variables $\mathbf{z}_{jL} \times \mathbf{z}_{kR}$ and $\mathbf{z}_{jR} \times \mathbf{z}_{kL}$ are identically associated to $\mathbf{y}$, and thus they are averaged to give $\mathbf{n}_{jk} = \frac{1}{2}(\mathbf{z}_{jL} \times \mathbf{z}_{kR} + \mathbf{z}_{kL} \times \mathbf{z}_{jR})$. Variable $\mathbf{z}_{kL} \times \mathbf{z}_{kR}$ is simply $\mathbf{n}_{kk}$. Variables $\mathbf{z}_{iL} \times \mathbf{z}_{kL}$ and $\mathbf{z}_{iR} \times \mathbf{z}_{kR}$ are identically associated to $\mathbf{y}$, and thus they are averaged to give $\mathbf{m}_{ik} = \frac{1}{2}(\mathbf{z}_{iL} \times \mathbf{z}_{kL} + \mathbf{z}_{iR} \times \mathbf{z}_{kR})$. Variables $\mathbf{z}_{jL} \times \mathbf{z}_{kL}$ and $\mathbf{z}_{jR} \times \mathbf{z}_{kR}$ are identically associated to $\mathbf{y}$, and thus they are averaged to give $\mathbf{m}_{jk} = \frac{1}{2}(\mathbf{z}_{jL} \times \mathbf{z}_{kL} + \mathbf{z}_{jR} \times \mathbf{z}_{kR})$. Variables $\mathbf{z}_{iL} \times \mathbf{z}_{jR} \times \mathbf{z}_{kL}$, $\mathbf{z}_{iL} \times \mathbf{z}_{jR} \times \mathbf{z}_{kR}$, $\mathbf{z}_{iR} \times \mathbf{z}_{jL} \times \mathbf{z}_{kL}$ and $\mathbf{z}_{iR} \times \mathbf{z}_{jL} \times \mathbf{z}_{kR}$ are identically associated to $\mathbf{y}$, and thus they are averaged to give $\frac{1}{4}(\mathbf{z}_{iL} \times \mathbf{z}_{jR} \times \mathbf{z}_{kL} + \mathbf{z}_{iL} \times \mathbf{z}_{jR} \times \mathbf{z}_{kR} + \mathbf{z}_{iR} \times \mathbf{z}_{jL} \times \mathbf{z}_{kL} + \mathbf{z}_{iR} \times \mathbf{z}_{jL} \times \mathbf{z}_{kR}) = \frac{1}{2}(\mathbf{z}_{iL} \times \mathbf{z}_{jR} + \mathbf{z}_{jL} \times \mathbf{z}_{iR}) \times \frac{1}{2}(\mathbf{z}_{kL} + \mathbf{z}_{kR}) = \mathbf{n}_{ij} \times \mathbf{m}_k$. Variables $\mathbf{z}_{iR} \times \mathbf{z}_{kL} \times \mathbf{z}_{kR}$ and $\mathbf{z}_{iL} \times \mathbf{z}_{kL} \times \mathbf{z}_{kR}$ are identically associated to $\mathbf{y}$, and thus they are averaged to give $\frac{1}{2}(\mathbf{z}_{iR} \times \mathbf{z}_{kL} \times \mathbf{z}_{kR} + \mathbf{z}_{iL} \times \mathbf{z}_{kL} \times \mathbf{z}_{kR}) = \frac{1}{2}(\mathbf{z}_{kL} \times \mathbf{z}_{kR} \times (\mathbf{z}_{iL} + \mathbf{z}_{iR})) = (\mathbf{z}_{kL} \times \mathbf{z}_{kR}) \times \frac{1}{2}(\mathbf{z}_{iL} + \mathbf{z}_{iR}) = \mathbf{n}_{kk} \times \mathbf{m}_i$. Variables $\mathbf{z}_{jR} \times \mathbf{z}_{kL} \times \mathbf{z}_{kR}$

and $\mathbf{z}_{jL} \times \mathbf{z}_{kL} \times \mathbf{z}_{kR}$ are identically associated to $\mathbf{y}$, and thus they are averaged to give $\frac{1}{2}(\mathbf{z}_{jR} \times \mathbf{z}_{kL} \times \mathbf{z}_{kR} + \mathbf{z}_{jL} \times \mathbf{z}_{kL} \times \mathbf{z}_{kR}) = \frac{1}{2}(\mathbf{z}_{kL} \times \mathbf{z}_{kR} \times (\mathbf{z}_{jL} + \mathbf{z}_{jR})) = (\mathbf{z}_{kL} \times \mathbf{z}_{kR}) \times \frac{1}{2}(\mathbf{z}_{jL} + \mathbf{z}_{jR}) = \mathbf{n}_{kk} \times \mathbf{m}_j$. These simplifications give a new set $\mathbf{C} = \{\mathbf{m}_i, \mathbf{m}_j, \mathbf{m}_k, \mathbf{m}_{ik}, \mathbf{m}_{jk}, \mathbf{n}_{ij}, \mathbf{n}_{jk}, \mathbf{n}_{ik}, \mathbf{n}_{kk}, \mathbf{m}_{jk}, \mathbf{n}_{ij} \times \mathbf{m}_k, \mathbf{n}_{kk} \times \mathbf{m}_i, \mathbf{n}_{kk} \times \mathbf{m}_j\}$ as confounder of $\mathbf{c}_{ijk}$.
