# Supplementary figures and images for: Uncovering direct and indirect molecular determinants of chromatin loops using a computational integrative approach
Source: PLoS Comput Biol. 2017 May 23;13(5):e1005538. doi: 10.1371/journal.pcbi.1005538 (PMC5462476; doi:10.1371/journal.pcbi.1005538)

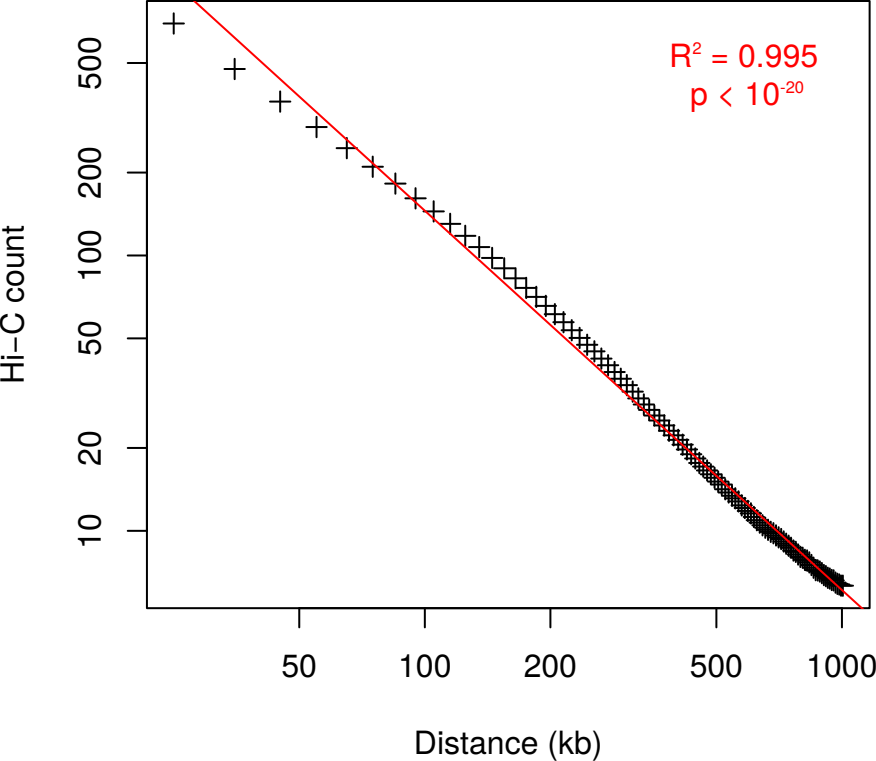

Supplement: S1 Fig — 20 kb resolution for distances comprised between 10kb and 1Mb. Drosophila Kc167 cell data. (PDF) [file pcbi.1005538.s002.pdf]

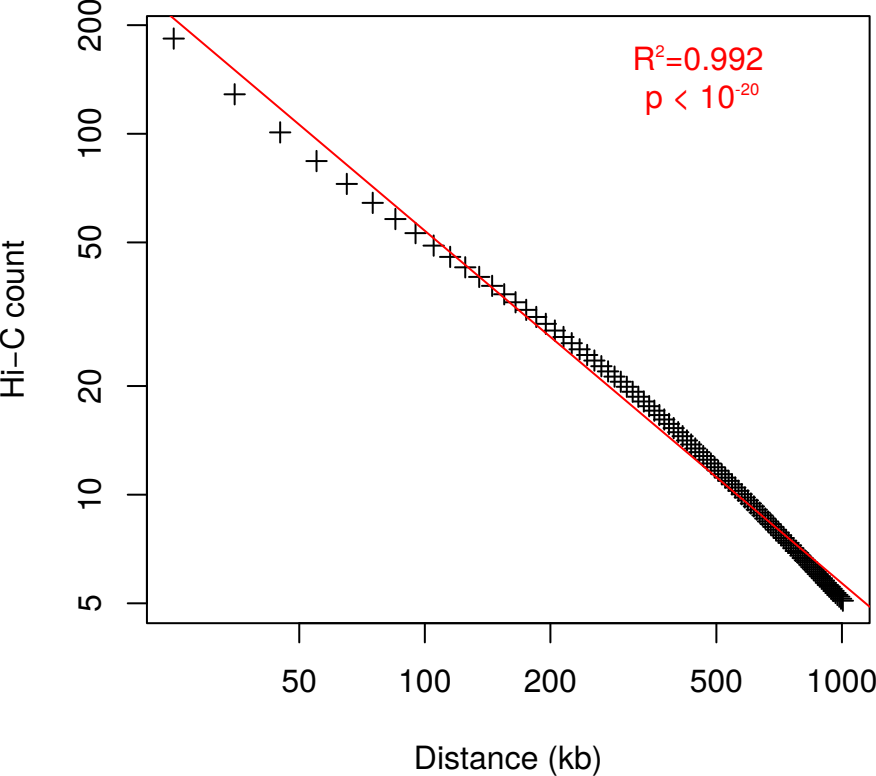

Supplement: S2 Fig — 20 kb resolution for distances comprised between 10kb and 1Mb. Human GM12878 cell data. (PDF) [file pcbi.1005538.s003.pdf]
