# Supplementary material for: Uncovering direct and indirect molecular determinants of chromatin loops using a computational integrative approach
Source: PLoS Comput Biol. 2017 May 23;13(5):e1005538. doi: 10.1371/journal.pcbi.1005538 (PMC5462476; doi:10.1371/journal.pcbi.1005538)
Supplement: S1 Table — Long-range contacts measured by homologous interaction variable betas. GM12878 cell ChIP-seq data. (PDF) [file pcbi.1005538.s004.pdf]

Feature	Beta	Standard Error	Z	p value	Feature	Beta	Standard Error	Z	p value
RAD21	89,48	6,18	14,49	1,39E-47	NRF1	72,97	23,38	3,12	0,0018018842
CTCF	37,14	5,40	6,88	6,19E-12	NRSF	5,02	13,78	0,36	0,7157716553
YY1	31,85	2,65	12,00	3,44E-33	P300	264,53	19,72	13,42	4,82E-41
ZBTB33	119,44	55,32	2,16	0,0308575886	PAX5	84,55	5,41	15,62	5,14E-55
MAZ	15,93	1,62	9,82	9,37E-23	PBX3	47,82	18,04	2,65	0,0080420037
JUND	258,45	42,25	6,12	9,50E-10	PML	22,53	1,01	22,30	3,95E-110
ZNF143	1,21	5,94	0,20	0,8384801062	POL2	0,07	0,02	3,56	0,0003728767
EZH2	120,11	33,52	3,58	0,0003386034	POL3	336,91	85,29	3,95	7,81E-05
ATF2	50,16	1,58	31,72	7,82E-221	POU2F2	67,91	3,25	20,90	5,38E-97
ATF3	-363,05	204,62	-1,77	0,0760263876	RFX5	25,66	19,54	1,31	0,1891393709
BATF	134,35	5,98	22,48	6,42E-112	RUNX3	31,71	0,97	32,81	4,80E-236
BCL11A	178,66	7,19	24,84	3,49E-136	RXRA	628,89	165,31	3,80	0,0001421424
BCL3	43,85	4,27	10,26	1,04E-24	SIN3A	27,86	2,20	12,67	8,71E-37
BCLAF1	77,49	8,68	8,93	4,33E-19	SIX5	190,03	30,74	6,18	6,32E-10
BHLHE40	80,18	5,79	13,85	1,23E-43	SMC3	75,41	5,36	14,08	5,12E-45
BRCA1	195,02	184,23	1,06	0,289808917	SP1	66,32	4,37	15,18	5,06E-52
CEBPB	99,84	6,30	15,84	1,59E-56	SPI1	70,24	3,95	17,77	1,16E-70
CFOS	148,73	48,06	3,09	0,0019687706	SRF	107,68	23,41	4,60	4,22E-06
CHD1	62,89	5,13	12,25	1,69E-34	STAT1	39,87	35,53	1,12	0,2617092007
CHD2	64,91	3,39	19,17	6,20E-82	STAT3	102,62	7,18	14,28	2,72E-46
CMYC	20,52	5,98	3,43	0,0005955262	STAT5	35,39	3,31	10,69	1,09E-26
COREST	90,03	33,21	2,71	0,0067081113	TBLR1	74,91	3,24	23,14	1,76E-118
E2F4	64,48	26,30	2,45	0,0142343307	TBP	74,01	3,14	23,54	1,71E-122
EBF1	36,60	2,85	12,85	8,47E-38	TCF12	87,82	6,15	14,28	2,95E-46
EGR1	87,91	8,05	10,92	8,84E-28	TCF3	59,26	5,44	10,90	1,21E-27
ELF1	31,02	2,55	12,16	5,26E-34	TR4	60,94	64,99	0,94	0,3484738337
ELK1	113,97	12,54	9,09	1,02E-19	USF1	31,89	13,57	2,35	0,0188014169
FOXM1	40,25	1,66	24,22	1,25E-129	USF2	77,78	13,52	5,75	8,70E-09
GABP	34,63	10,52	3,29	0,0009936862	WHIP	41,66	2,29	18,22	3,37E-74
IKZF1	39,35	2,13	18,51	1,65E-76	ZEB1	109,89	17,22	6,38	1,74E-10
IRF4	104,27	5,99	17,42	5,86E-68	ZNF274	104,69	32,85	3,19	0,0014396383
MAX	34,20	2,95	11,61	3,67E-31	ZZZ3	119,91	97,79	1,23	0,2201545944
MEF2C	189,33	13,71	13,81	2,23E-43	DNase	5,04	0,77	6,57	5,04E-11
MTA3	22,43	1,21	18,51	1,86E-76	H3K27me3	0,05	0,00	21,81	2,03E-105
MXI1	33,10	1,92	17,24	1,23E-66	H3K36me3	0,11	0,00	43,89	0
NFATC1	1,07	0,64	1,67	0,095464965	H3K4me2	0,36	0,01	47,41	0
NFE2	264,56	264,39	1,00	0,3169943492	H3K4me3	0,21	0,01	34,70	6,94E-264
NFIC	27,91	0,91	30,83	1,11E-208	H3K9ac	0,28	0,01	28,99	1,00E-184
NFKB	80,26	3,39	23,68	6,36E-124	H3K9me3	0,07	0,00	19,14	1,13E-81
NFYA	160,82	46,03	3,49	0,0004761013	H4K20me1	-0,07	0,00	-21,20	8,79E-100
NFYB	29,44	5,60	5,26	1,43E-07					
