# Supplementary material for: Uncovering direct and indirect molecular determinants of chromatin loops using a computational integrative approach
Source: PLoS Comput Biol. 2017 May 23;13(5):e1005538. doi: 10.1371/journal.pcbi.1005538 (PMC5462476; doi:10.1371/journal.pcbi.1005538)
Supplement: S2 Table — Mediating effect of cohesin measured by homologous interaction cofactor variable betas. GM12878 cell ChIP-seq data. (PDF) [file pcbi.1005538.s005.pdf]

Feature	Beta	Standard Error	Z	p value	Feature	Beta	Standard Error	Z	p value
POLII	446,62	55,18	8,09	5,77E-16	NFKB	4266,55	4268,74	1,00	0,3175593529
ELF1	22382,19	3271,83	6,84	7,87E-12	STAT1	36520,50	38003,17	0,96	0,336559362
YY1	22524,33	3514,55	6,41	1,47E-10	CHD2	4183,39	4393,45	0,95	0,3410015284
EGR1	73961,62	11773,47	6,28	3,34E-10	MTA3	1637,72	1985,35	0,82	0,4094260183
ZNF143	48199,37	8034,42	6,00	1,98E-09	NFYB	8268,64	10184,81	0,81	0,4168720154
NRF1	175736,43	34997,61	5,02	5,13E-07	ZZZ3	351790,18	500256,15	0,70	0,481918589
CTCF	40884,49	8807,24	4,64	3,45E-06	RXRA	129170,30	192226,11	0,67	0,5016024132
FOXM1	9139,85	2040,80	4,48	7,51E-06	CMYC	5384,58	8360,91	0,64	0,5195634916
MXI1	10248,83	2471,92	4,15	3,38E-05	NFATC1	1727,64	2790,74	0,62	0,5358760602
RUNX3	5210,66	1344,68	3,88	0,0001066216	TCF3	3915,82	6946,77	0,56	0,572964987
MAZ	7112,56	1906,78	3,73	0,0001913683	BHLHE40	3858,72	6964,75	0,55	0,5795546754
SIN3A	10670,22	3142,48	3,40	0,000685096	BRCA1	441710,25	806999,75	0,55	0,5841392047
SPI1	21711,57	6494,64	3,34	0,0008287829	BCLAF1	4361,81	9812,66	0,44	0,6566749083
TCF12	18974,12	5962,38	3,18	0,0014610791	CFOS	29926,29	74367,69	0,40	0,6873824438
PAX5	22147,22	7160,22	3,09	0,0019808179	P300	8223,99	24063,61	0,34	0,7325311933
SP1	17274,64	5710,63	3,02	0,0024863553	BATF	2736,19	8070,72	0,34	0,7345890491
SMC3	20241,88	6944,86	2,91	0,0035607959	WHIP	690,52	3504,07	0,20	0,8437793206
BCL11A	19747,65	8267,58	2,39	0,0169143244	USF1	3282,49	20780,49	0,16	0,874488028
NFIC	2288,67	1052,46	2,17	0,0296612828	TR4	24308,94	154003,34	0,16	0,8745774885
SIX5	124288,10	57372,55	2,17	0,030285684	CHD1	824,06	5305,97	0,16	0,8765783063
COREST	104492,40	49449,45	2,11	0,0345908711	ELK1	1967,37	16134,18	0,12	0,902948308
GABP	42864,91	20912,43	2,05	0,0403904145	USF2	1592,81	16292,40	0,10	0,9221195189
PML	2940,78	1486,63	1,98	0,0479113953	RFX5	-495,02	27761,54	-0,02	0,9857736588
IRF4	15199,04	7780,41	1,95	0,0507601613	CEBPB	-587,26	6404,57	-0,09	0,926941231
SRF	59167,47	31069,64	1,90	0,0568646009	ZBTB33	-14396,21	81230,59	-0,18	0,8593305291
EBF1	6499,53	3495,63	1,86	0,0629804347	IKZF1	-798,28	2704,12	-0,30	0,7678340382
E2F4	65206,49	38774,70	1,68	0,0926316174	NRSF	-9102,84	27394,61	-0,33	0,7396734389
TBLR1	5758,27	3494,50	1,65	0,0993914431	ATF2	-723,49	2044,51	-0,35	0,7234363032
MAX	5260,17	3601,44	1,46	0,1441324624	NFE2	-181151,01	380746,24	-0,48	0,6342319381
TBP	4753,10	3495,02	1,36	0,1738416731	ZEB1	-12289,06	22166,11	-0,55	0,5792998161
ATF3	433472,61	339093,77	1,28	0,2011342696	POLIII	-182705,16	289915,85	-0,63	0,5285632996
JUND	83582,09	67039,40	1,25	0,2124852653	BCL3	-6051,65	5791,97	-1,04	0,2960997441
STAT3	10361,88	9286,49	1,12	0,2645069823	EZH2	-56091,12	52764,20	-1,06	0,2877580917
POU2F2	4532,67	4309,43	1,05	0,292889953	ZNF274	-6103,66	5380,66	-1,13	0,2566392643
MEF2C	15883,06	15172,83	1,05	0,2951876293	STAT5	-5657,97	4476,49	-1,26	0,2062553593
NFYA	74513,38	73082,85	1,02	0,3079305248	PBX3	-58965,91	25576,16	-2,31	0,0211384225
