# Supplementary material for: Uncovering direct and indirect molecular determinants of chromatin loops using a computational integrative approach
Source: PLoS Comput Biol. 2017 May 23;13(5):e1005538. doi: 10.1371/journal.pcbi.1005538 (PMC5462476; doi:10.1371/journal.pcbi.1005538)
Supplement: S3 Table — GM12878 cell ChIP-seq data. (PDF) [file pcbi.1005538.s006.pdf]

List of cliques from the attraction graph					
ATF2/NRF1/SIN3A	CHD1/CMYC/RXRA	EBF1/WHIP/H3K9ac	GABP/WHIP/H3K36me3	MXI1/H3K36me3/H3K4me2	RFX5/H3K4me2/H3K9me3
ATF3/FOXM1/H3K9ac	CHD1/E2F4/P300	ELF1/H3K36me3/H3K4me2	GABP/WHIP/H3K4me3	NFATC1/SMC3/ZNF143	RUNX3/H3K4me2/H3K9me3
ATF3/GABP/TBP	CHD1/E2F4/STAT3	ELF1/H3K36me3/promoterH3K4me3	GABP/H3K36me3/H3K4me3	NFATC1/TR4/ZNF143	SIN3A/TBP/promoterH3K4me3
ATF3/GABP/promoterH3K4me3	CHD1/E2F4/H3K36me3	ELF1/H3K4me2/promoterH3K4me3	GABP/H3K36me3/promoterH3K4me3	NFE2/RAD21/H3K9me3	SIN3A/H3K36me3/promoterH3K4me3
ATF3/IKZF1/H3K9ac	CHD1/E2F4/H3K9me3	ELK1/EZH2/IKZF1	IKZF1/NFYB/enhancer	NFE2/ZNF274/H3K9me3	STAT1/ZNF274/H3K9me3
ATF3/IKZF1/H4K20me1	CHD1/RAD21/H3K9ac	EZH2/IKZF1/RFX5	IKZF1/POL2/RFX5	NFKB/STAT3/TBLR1	TR4/ZNF143/ZZZ3
ATF3/RAD21/H3K9ac	CHD1/RAD21/H3K9me3	EZH2/IKZF1/STAT1	IKZF1/POL2/H3K9ac	NFYA/H3K36me3/promoterH3K4me3	WHIP/H3K36me3/H3K4me3
ATF3/TBP/promoterH3K4me3	CHD1/RXRA/STAT1	EZH2/IKZF1/STAT3	IKZF1/POL2/enhancer	NRF1/SIN3A/TBP	WHIP/H3K4me3/H3K9ac
ATF3/TR4/H4K20me1	CHD1/RXRA/H3K36me3	EZH2/IKZF1/ZNF274	IKZF1/RFX5/STAT3	NRF1/H3K4me2/H3K4me3	H3K36me3/H3K4me2/H3K4me3
BATF/NFE2/PBX3	CHD1/STAT1/H3K9me3	EZH2/P300/ZZZ3	IKZF1/STAT1/ZNF274	NRF1/H3K4me2/enhancer	H3K36me3/H3K4me2/promoterH3K4me3
BATF/NFE2/H3K9me3	CHD1/STAT3/H3K9me3	EZH2/RFX5/STAT3	IRF4/POL2/RFX5	P300/SRF/STAT5	H3K4me2/H3K4me3/H3K9me3
BATF/PML/promoterH3K4me3	CHD1/H3K36me3/H3K4me3	EZH2/RFX5/TR4	IRF4/POL2/enhancer	P300/SRF/TCF3	ATF3/GABP/TBP/promoterH3K4me3
BCL11A/NFE2/TR4	CHD1/H3K4me3/H3K9ac	EZH2/STAT1/ZNF143	IRF4/RFX5/STAT3	PAX5/POL3/H3K4me3	BCLAF1/PML/H3K4me3/H3K9ac
BCL3/EBF1/WHIP	CHD1/H3K4me3/H3K9me3	EZH2/STAT1/ZNF274	JUND/MEF2C/POU2F2	PAX5/POL3/H3K9ac	BRCA1/EZH2/TR4/ZZZ3
BCL3/EBF1/H4K20me1	CHD2/MEF2C/TCF12	EZH2/TR4/ZNF143	JUND/POL2/SPI1	PAX5/H3K4me2/H3K4me3	CHD1/E2F4/STAT3/H3K9me3
BCL3/MAZ/H4K20me1	CMYC/NFYB/ZZZ3	EZH2/TR4/ZZZ3	JUND/POL2/enhancer	PAX5/H3K4me3/H3K9ac	ELF1/H3K36me3/H3K4me2/promoterH3K4me3
BCL3/RFX5/WHIP	CMYC/PBX3/ZBTB33	EZH2/ZNF143/ZZZ3	JUND/POU2F2/enhancer	PBX3/POL3/RUNX3	EZH2/IKZF1/RFX5/STAT3
BCLAF1/BRCA1/H3K4me3	CMYC/PML/promoterH3K4me3	FOXM1/NFYB/SIN3A	JUND/H3K4me2/enhancer	PBX3/RUNX3/H3K4me2	EZH2/IKZF1/STAT1/ZNF274
BCLAF1/PML/STAT1	CTCF/FOXM1/SMC3	FOXM1/NFYB/enhancer	MAX/MAZ/STAT1	PML/H3K36me3/H3K4me3	EZH2/TR4/ZNF143/ZZZ3
BCLAF1/PML/H3K4me3	CTCF/FOXM1/H3K9ac	FOXM1/PML/SMC3	MAX/POL2/H3K4me3	PML/H3K36me3/promoterH3K4me3	GABP/PML/H3K36me3/H3K4me3
BCLAF1/PML/H3K9ac	CTCF/FOXM1/enhancer	FOXM1/PML/H3K9ac	MAX/WHIP/H3K36me3	PML/H3K4me3/H3K9ac	GABP/PML/H3K36me3/promoterH3K4me3
BCLAF1/RXRA/STAT1	CTCF/IKZF1/STAT3	FOXM1/POL2/H3K9ac	MAX/WHIP/H3K4me3	POL2/SPI1/H3K9ac	GABP/POL3/TBP/promoterH3K4me3
BCLAF1/H3K4me3/H3K9ac	CTCF/IKZF1/H3K9ac	FOXM1/POL2/enhancer	MAX/H3K36me3/H3K4me3	POL2/H3K4me3/H3K9ac	GABP/POL3/WHIP/H3K4me3
BHLHE40/NFKB/TBLR1	CTCF/IKZF1/enhancer	GABP/P300/POL3	MEF2C/MTA3/POL3	POL3/RAD21/H3K9ac	GABP/SIN3A/TBP/promoterH3K4me3
BRCA1/EZH2/TR4	CTCF/RAD21/SMC3	GABP/PML/H3K36me3	MEF2C/POL3/RUNX3	POL3/RUNX3/H3K9ac	GABP/SIN3A/H3K36me3/promoterH3K4me3
BRCA1/EZH2/ZZZ3	CTCF/RAD21/H3K9ac	GABP/PML/H3K4me3	MEF2C/POL3/WHIP	POL3/TBP/promoterH3K4me3	GABP/WHIP/H3K36me3/H3K4me3
BRCA1/TR4/ZZZ3	CTCF/H3K4me2/H3K4me3	GABP/PML/promoterH3K4me3	MEF2C/POL3/H3K4me3	POL3/WHIP/H3K4me3	MAX/WHIP/H3K36me3/H3K4me3
BRCA1/H3K36me3/H3K4me3	CTCF/H3K4me2/enhancer	GABP/POL3/TBP	MEF2C/POU2F2/H3K4me3	POL3/WHIP/H3K9ac	MEF2C/POL3/WHIP/H3K4me3
CEBPB/CTCF/enhancer	CTCF/H3K4me3/H3K9ac	GABP/POL3/WHIP	MEF2C/POU2F2/H3K9me3	POL3/H3K4me3/H3K9ac	MEF2C/POU2F2/H3K4me3/H3K9me3
CEBPB/NRF1/enhancer	E2F4/NFYA/H3K36me3	GABP/POL3/H3K4me3	MEF2C/RUNX3/H3K9me3	POU2F2/SP1/enhancer	PAX5/POL3/H3K4me3/H3K9ac
CFOS/E2F4/P300	E2F4/P300/STAT5	GABP/POL3/promoterH3K4me3	MEF2C/WHIP/H3K4me3	POU2F2/H3K4me3/H3K9me3	POL3/WHIP/H3K4me3/H3K9ac
CFOS/E2F4/TBP	E2F4/P300/TCF3	GABP/SIN3A/TBP	MEF2C/H3K4me3/H3K9me3	RFX5/STAT3/ZEB1	
CFOS/E2F4/ZBTB33	E2F4/PML/H3K36me3	GABP/SIN3A/H3K36me3	MTA3/POL3/H3K9ac	RFX5/STAT3/H3K9me3	
CFOS/E2F4/enhancer	E2F4/STAT3/ZBTB33	GABP/SIN3A/promoterH3K4me3	MTA3/STAT1/ZBTB33	RFX5/USF1/ZEB1	
CFOS/P300/ZZZ3	E2F4/STAT3/H3K9me3	GABP/TBP/promoterH3K4me3	MXI1/NFYA/H3K36me3	RFX5/ZEB1/H3K4me2	
